# Lysosomal and network alterations in human mucopolysaccharidosis type VII iPSC-derived neurons

**DOI:** 10.1038/s41598-018-34523-3

**Published:** 2018-11-09

**Authors:** Neus Bayó-Puxan, Ana Paula Terrasso, Sophie Creyssels, Daniel Simão, Christina Begon-Pescia, Marina Lavigne, Sara Salinas, Florence Bernex, Assumpció Bosch, Vasiliki Kalatzis, Thierry Levade, Ana Maria Cuervo, Philippe Lory, Antonella Consiglio, Catarina Brito, Eric J. Kremer

**Affiliations:** 1 Institute de Génétique Moléculaire de Montpellier, University of Montpellier, CNRS, Montpellier, France; 2 Department of Pathology and Experimental Therapeutics, Bellvitge University Hospital-IDIBELL, Institute of Biomedicine of the University of Barcelona (IBUB), Barcelona, Spain; 3 iBET - Instituto de Biologia Experimental e Tecnológica, Oeiras, Portugal; 4 Instituto de Tecnologia Química e Biológica António Xavier, Universidade Nova de Lisboa, Oeiras, Portugal; 5 IRCM, Inserm, University Montpellier, Montpellier, France; 6 Departament Bioquímica i Biologia Molecular, and Center of Animal Biotechnology and Gene Therapy (CBATEG), Universitat Autònoma Barcelona, Bellaterra, Spain; 7 INM, Inserm, University Montpellier, Montpellier, France; 8 Laboratoire de Biochimie Métabolique, IFB, CHU Purpan, and Inserm 1037, CRCT, University Paul Sabatier Toulouse-III, Toulouse, France; 9 Department of Developmental and Molecular Biology and Institute for Aging Studies, Albert Einstein College of Medicine, Bronx, NY USA; 10 IGF, CNRS, Inserm, University Montpellier, Montpellier, France; 11 Department of Molecular and Translational Medicine, University of Brescia, Brescia, BS Italy; 12 The Discoveries Centre for Regenerative and Precision Medicine, NOVA University Lisbon, Av da República, 2780-157 Oeiras Portugal

## Abstract

Mucopolysaccharidosis type VII (MPS VII) is a lysosomal storage disease caused by deficient β-glucuronidase (β-gluc) activity. Significantly reduced β-gluc activity leads to accumulation of glycosaminoglycans (GAGs) in many tissues, including the brain. Numerous combinations of mutations in *GUSB* (the gene that codes for β-gluc) cause a range of neurological features that make disease prognosis and treatment challenging. Currently, there is little understanding of the molecular basis for MPS VII brain anomalies. To identify a neuronal phenotype that could be used to complement genetic analyses, we generated two iPSC clones derived from skin fibroblasts of an MPS VII patient. We found that MPS VII neurons exhibited reduced β-gluc activity and showed previously established disease-associated phenotypes, including GAGs accumulation, expanded endocytic compartments, accumulation of lipofuscin granules, more autophagosomes, and altered lysosome function. Addition of recombinant β-gluc to MPS VII neurons, which mimics enzyme replacement therapy, restored disease-associated phenotypes to levels similar to the healthy control. MPS VII neural cells cultured as 3D neurospheroids showed upregulated *GFAP* gene expression, which was associated with astrocyte reactivity, and downregulation of GABAergic neuron markers. Spontaneous calcium imaging analysis of MPS VII neurospheroids showed reduced neuronal activity and altered network connectivity in patient-derived neurospheroids compared to a healthy control. These results demonstrate the interplay between reduced β-gluc activity, GAG accumulation and alterations in neuronal activity, and provide a human experimental model for elucidating the bases of MPS VII-associated cognitive defects.

## Introduction

Lysosomal storage disorders (LSD) are caused by intra- and extracellular accumulation of undigested macromolecules that induce dysfunction of the greater lysosomal system. Among LSD, mucopolysaccharidoses (MPS) are caused by deficiency in enzymatic activities that degrade glycosaminoglycans (GAGs). GAGs are the most abundant polysaccharides of the extracellular matrix (ECM) and, with the exception of hyaluronic acid, are covalently attached to protein moieties to form proteoglycans^1^. β-glucuronidase (β-gluc, EC 3.2.1.31), is found in lysosomes of all nucleated mammalian cell and is involved in the step-wise degradation of GAGs by removing glucuronic acid residues. Impaired β-gluc activity results in partial degradation and accumulation of chondroitin sulfate, dermatan sulfate and heparan sulfate GAGs.

MPS type VII (MPS VII), a neuronopathic form of an MPS, is an ultra-rare disease with an estimated frequency of ~1:2 000 000^2^. It has an autosomal recessive inheritance pattern caused by mutations in *GUSB gene*^3^. There are at least 49 disease-associated mutations that contribute to hepatosplenomegaly, cardiac valvular abnormalities, recurrent pulmonary infections, growth retardation, mobility problems, dysostosis multiplex, facial dysmorphia, visual and hearing defects, cognitive defects and/or early death^4^. Most of the ~100 identified MPS VII patients present limited vocabulary and mental retardation^2^. The most severe phenotype is *hydrops fetalis*, while a mild phenotype displays a late onset and near normal intelligence^4^. Notably, MPS VII patients with similar mutations can have variable levels of cognitive impairment, which suggests that other factors influence disease severity and confound reliable prediction of disease progression. While most mutations underlying MPS VII are known, the mechanistic links between reduced β-gluc activity, GAG accumulation and neurological anomalies are just beginning to be addressed^2,5^. Human MPS VII brain cell models could contribute to understand how defects in the greater lysosomal system cause progressive (instead of immediate) brain impairment/dysfunction and may also inspire novel therapies.

Using induced pluripotent stem cell (iPSC) technology^6,7^, a handful of LSD cell models have been established (reviewed in^8^), including Gaucher^9^, Hurler^10^, Pompe^11^, Sanfilippo B and C^12,13^ and Niemann-Pick type C1^14^. These patient-derived iPSCs were differentiated in 2D cultures into several cell types, including brain cells, and recapitulated morphological, biochemical and/or functional hallmarks of the disease. LSD patient iPSCs engineered to overexpress functional enzymes have been also reported^15–17^. While clearly informative, these cell models lack the dynamic events linked to interactions with proteoglycans and hyaluronic acid, the main components of brain ECM. This may impact the interactions between neural cells and ECM that regulate neural cell fate and functionality^18^. Thus, there is a need for MPS brain cell models in which the ECM and its dynamic remodeling are taken into account. We recently developed a 3D culture strategy, using stirred-tank bioreactors, to differentiate iPSC-derived neural precursor cells (iPSC-NPCs) into neurons, astrocytes and oligodendrocytes. This strategy allows cells to produce an ECM, fostering cell-cell and cell-ECM interactions that recapitulate many facet of brain cell architecture^19,20^. Using the classic 2D cultures and 3D neurospheroid cultures, we set out to test whether MPS VII iPSC-NPCs and mature neurons could be used to understand disease-associated neurological anomalies. We show that MPS VII neurons differentiated in 2D and 3D have elevated GAG content, expanded endocytic compartment, impaired membrane receptor lysosome-mediated degradation and altered functional connectivity. The use of MPS VII neurospheroids cultured in stirred-tank bioreactors allowed us to identify cellular features that likely impact brain-associated anomalies.

## Results

### Generation and characterization of human MPS VII iPSC

**Figure 1:**
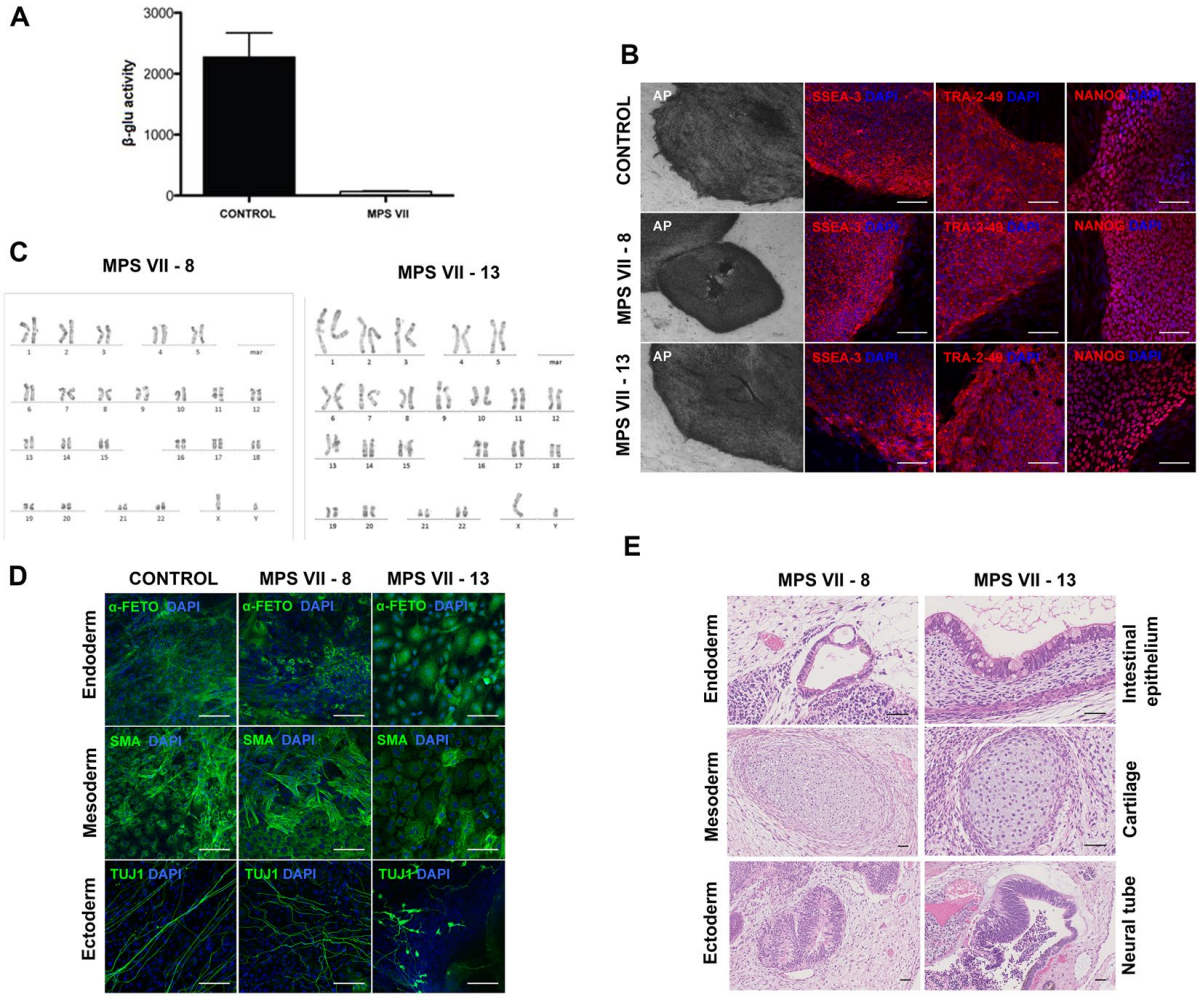
Generation and characterization of human MPS VII iPSC. (**A**) β-gluc enzymatic activity (expressed in nmol 4-MU/μg of protein/h) in control and MPS VII patient’s fibroblasts. (**B**) Representative colonies of control and MPS VII iPSC stained for alkaline phosphatase (AP) and the pluripotency-associated markers SSEA-3, Tra-2-49 and NANOG (all in red) and DAPI (blue); scale bars 200 µm (AP) and 100 µm (SSEA-3, Tra-2-49 and NANOG). (**C**) Karyotype of MPS VII iPSC. (**D**) Immunofluorescence microscopy of control and MPS VII iPSC differentiated *in vitro* and stained for the endoderm, mesoderm and ectoderm markers α-fetoprotein (green), smooth muscle actin (SMA, green) and βIII-tubulin (Tuj1, green), respectively; scale bars 100 µm. (**E**) Control and MPS VII iPSC differentiated *in vivo* by teratoma formation, stained with hematoxylin and eosin, showing potential to differentiate into endoderm (intestinal epithelium), mesoderm (cartilage) and ectoderm (neural tube); scale bars 100 µm.

Due to the dearth of MPS VII patients, we generated iPSCs from dermal fibroblasts of an MPS VII patient who was diagnosed at 3 years old based on decreased β-gluc activity and increased GAG storage^21^. The patient’s homozygous point mutations, which induced a Leu to Phe change at amino acid 176 in β-gluc was consistent with a severe MPS VII phenotype^4,21^.At the time of skin biopsy, the MPS VII patient was 27 years old and had severe mental retardation. Primary cultures of dermal fibroblasts were established and had approximately ≤2% of β-gluc activity compared to the fibroblasts from the healthy individual (Fig. 1A). Fibroblasts were reprogrammed at passages 2–4 with retroviral vector-mediated production of OCT4, cMYC, SOX2 and KLF4^7^. Following 3–4 weeks of culture, 20 compact PSC-like colonies emerged. These colonies were mechanically selected, expanded for at least 10 passages and tested for the expression of pluripotency markers.

We characterized two iPSC clones (^#^8 and ^#^13) independently. iPSC clones from healthy individuals (a gift from the Institute for Stem Cell Therapy and Exploration of Monogenic diseases, France) were used as controls. Expression of pluripotency-associated transcription factors (LIN28, OCT4, SOX2 and NANOG) and silencing of retroviral transgenes (OCT4, cMYC, SOX2 and KLF4) were confirmed by qRT-PCR. Expression of alkaline phosphatase (detected using anti-TRA-2-49) and its activity (Fig. 1B), the transcription factor NANOG, and SSEA-3 (stage-specific embryonic antigen 3) (Fig. 1B) are consistent with pluripotency. The MPS VII iPSC clones had a normal karyotype after more than 20 passages (Fig. 1C). The ability of iPSC to differentiate into the three different germ layers was assessed by *in vitro* embryoid body (EB) formation and *in vivo* teratoma formation. After EB formation, expression of tissue-specific markers for mesoderm (α-smooth muscle actin), endoderm (α-fetoprotein) and ectoderm (βIII-tubulin) were demonstrated by immunofluorescence analyses (Fig. 1D). The presence of germ layers derivatives was also confirmed in teratomas by hematoxylin and eosin staining and histological analyses (Fig. 1E). Together these data demonstrate that these clones harbored characteristics indicative of *bona fide* iPSCs.

### Generation and characterization of human MPS VII iPSC-NPC

**Figure 2:**
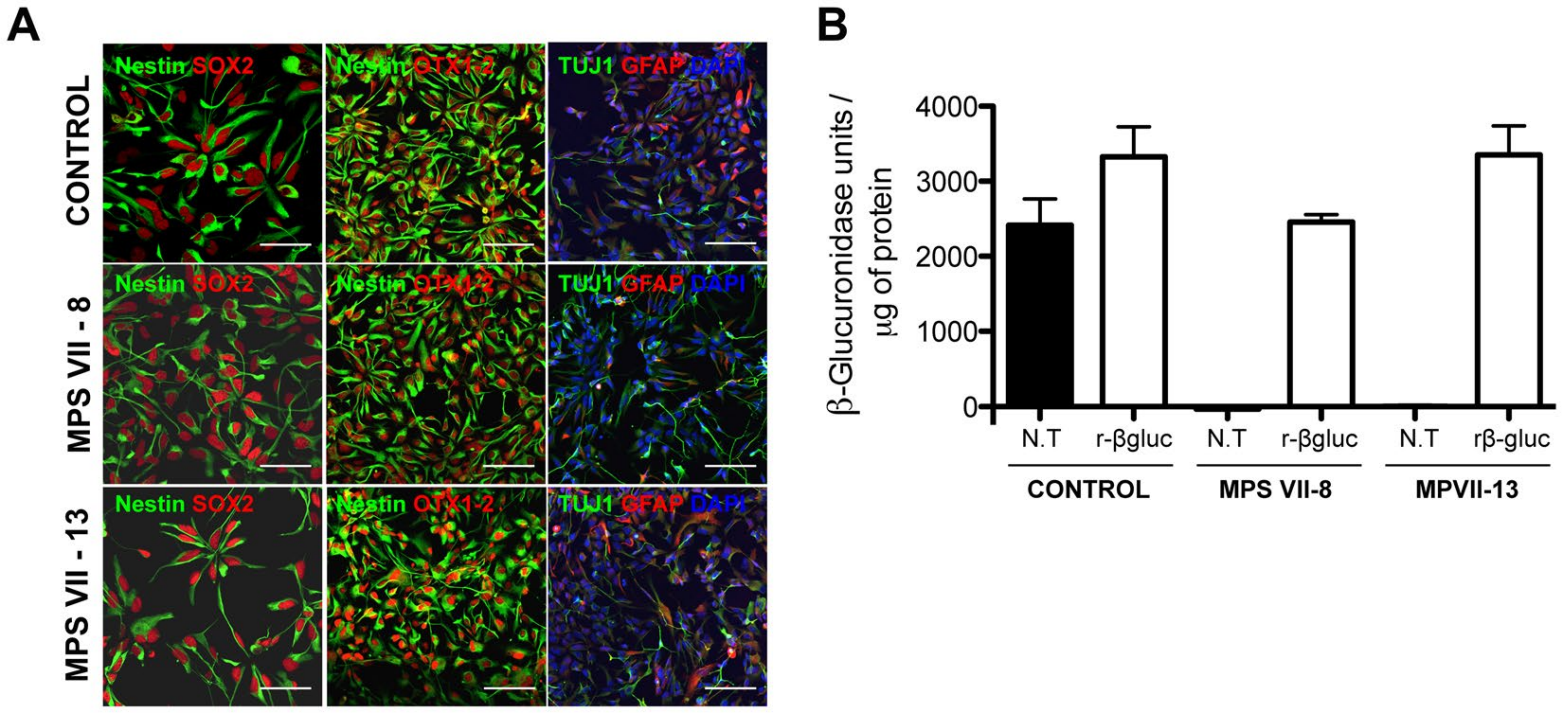
Characterization of human MPS VII iPSC-NPCs in 2D cultures. (**A**) Immunofluorescence microscopy of control and MPS VII iPSC-NPCs stained for NPC markers nestin (green) and SOX2 (red; first panel), scale bars 50 µm; nestin (green) and forebrain and midbrain progenitor marker OTX1/2 (red; second panel; and neuronal and astrocytic βIII-tubulin (Tuj1, green) and GFAP (red), respectively, DAPI (blue; third panel), scale bars 100 µm. (**B**) β-gluc enzymatic activity (expressed in nmol 4-MU/μg of protein/h) in control and MPS VII iPSC-NPC, non-treated (N.T.) and treated with recombinant β-gluc (rβ-gluc).

iPSC-NPCs were differentiated from control and MPS VII iPSCs by the dual SMAD inhibition protocol^22^. This involves induction of neuroepithelial cell (NEP)-rosettes from iPSC and NPC generation. NEP-rosettes appeared 8–12 days after induction and a homogeneous, expandable and phenotypically stable NPC population, as judged by the uniform co-expression of the neural progenitor markers Nestin and SOX2, was obtained after few passages (Fig. 2A). Expression of the transcription factor OTX-1/2 was also consistent with forebrain and midbrain NPCs (Fig. 2A). Early neuronal and astrocytic lineage markers (βIII-tubulin and GFAP), suggestive of the potential of NPCs to differentiate into neurons and astrocytes, were also detected (Fig. 2A). NPCs generated from control and MPS VII iPSC clones showed self-renewal capability and ability to generate cells with neuron-like morphology for at least 18 passages, indicating they were *bona fide* NPCs. No differences in self-renewal capability or viability were observed between NPCs derived from healthy and MPS VII iPSCs (Sup. Fig. 1).

Of note, a stromal feeder-free system was used to differentiate iPSC into NEP-rosettes and NPCs. Therefore, there was no exogenous β-gluc in the media, demonstrating that β-gluc deficiency did not interfere in the first stages of neuroectodermal differentiation. β-gluc activity in MPS VII NPCs was 50-fold lower than that in control NPC (Fig. 2B), confirming deficient β-gluc activity, and in line with that observed for MPS VII fibroblasts (Fig. 1A). To mimic enzyme replacement therapy (ERT), recombinant β-gluc (rβ-gluc) was added to NPC cultures. The addition of rβ-gluc led to increased β-gluc activity in MPS VII NPCs that was similar to that of control NPCs (Fig. 2B). Together these data demonstrate that NPCs with decreased β-gluc activity were readily generated from MPS VII iPSCs.

### Neural cells differentiated from MPS VII iPSC-NPCs recapitulate disease features

**Figure 3:**
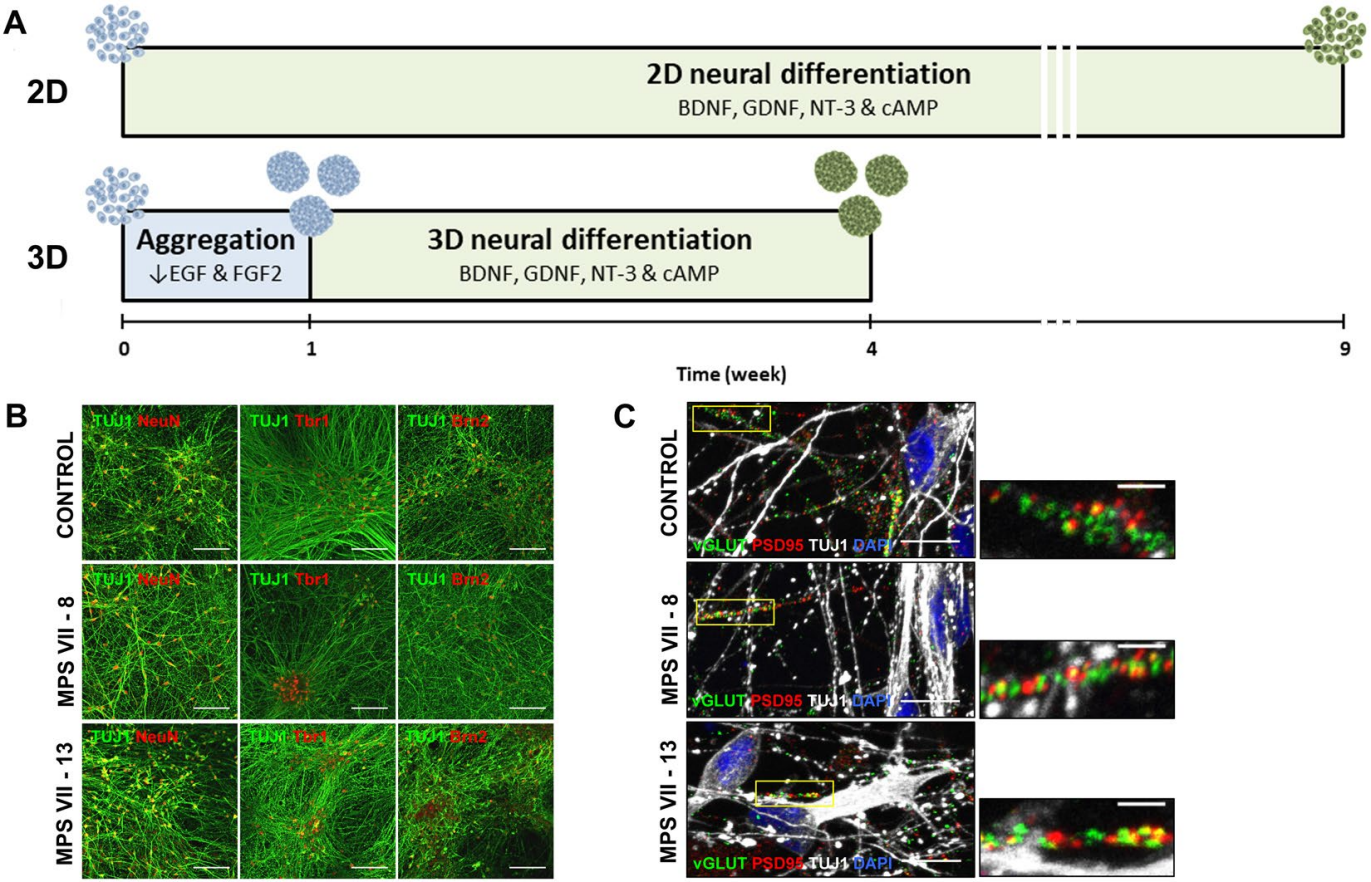
Schematic experimental workflow for differentiation of neural cells from control and MPS VII iPSC-NPCs in 2D (top) and 3D (bottom) culture systems and characterization of human MPS VII neurons in 2D cultures. (**A**) For 2D differentiation, iPSC-NPCs were plated and neural differentiation was induced by exposure to BDNF, GDNF, NT-3 and cAMP for 9 weeks. For 3D differentiation iPSC-NPCs were inoculated in a stirred suspension culture system, with reduced concentrations of growth factors EGF and FGF, and aggregated for 1 week, followed by induction of neural differentiation with BDNF, GDNF, NT-3 and cAMP for 3 weeks. (**B**) Representative images of control and MPS VII neurons stained for βIII-tubulin (Tuj1, green) and NeuN or cortical markers Tbr1 or Brn2 (red), respectively in first, second and third panels, scale bars 100 µm; (**C**) pre-synaptic protein vGLUT1 (green), post-synaptic protein PSD95 (red), βIII-tubulin (Tuj1, grey) and DAPI (blue), scale bars 100 µm; inserts (right) show synapsis formation at higher magnification, scale bars 5 μm.

To identify possible defects in MPS VII brain cells, control and MPS VII NPCs were further differentiated using 2D and 3D culture strategies (Fig. 3A), as these approaches could impact neural differentiation and cellular functions. For 2D differentiation, NPCs were cultured in neuronal induction medium (NIM) and differentiated into neurons within 3 weeks as determined by the expression of the neuronal markers βIII-tubulin and NeuN (Fig. 3B). Moreover, Tbr1^+^ and Brn2^+^ neurons were observed, indicating the capacity of MPS VII NPCs to give rise to neurons with cortical characteristics (Fig. 3B). Pre-synaptic (vGLUT1) and post-synaptic (PSD95) protein co-localization, suggestive of synapse formation and neuronal maturation, was achieved 7 weeks post-differentiation (Fig. 3C). iPSC-derived neurons presented membrane potentials of −50 mV and had voltage-dependent K^+^ channels consistent with functional synapses (not shown).

**Figure 4:**
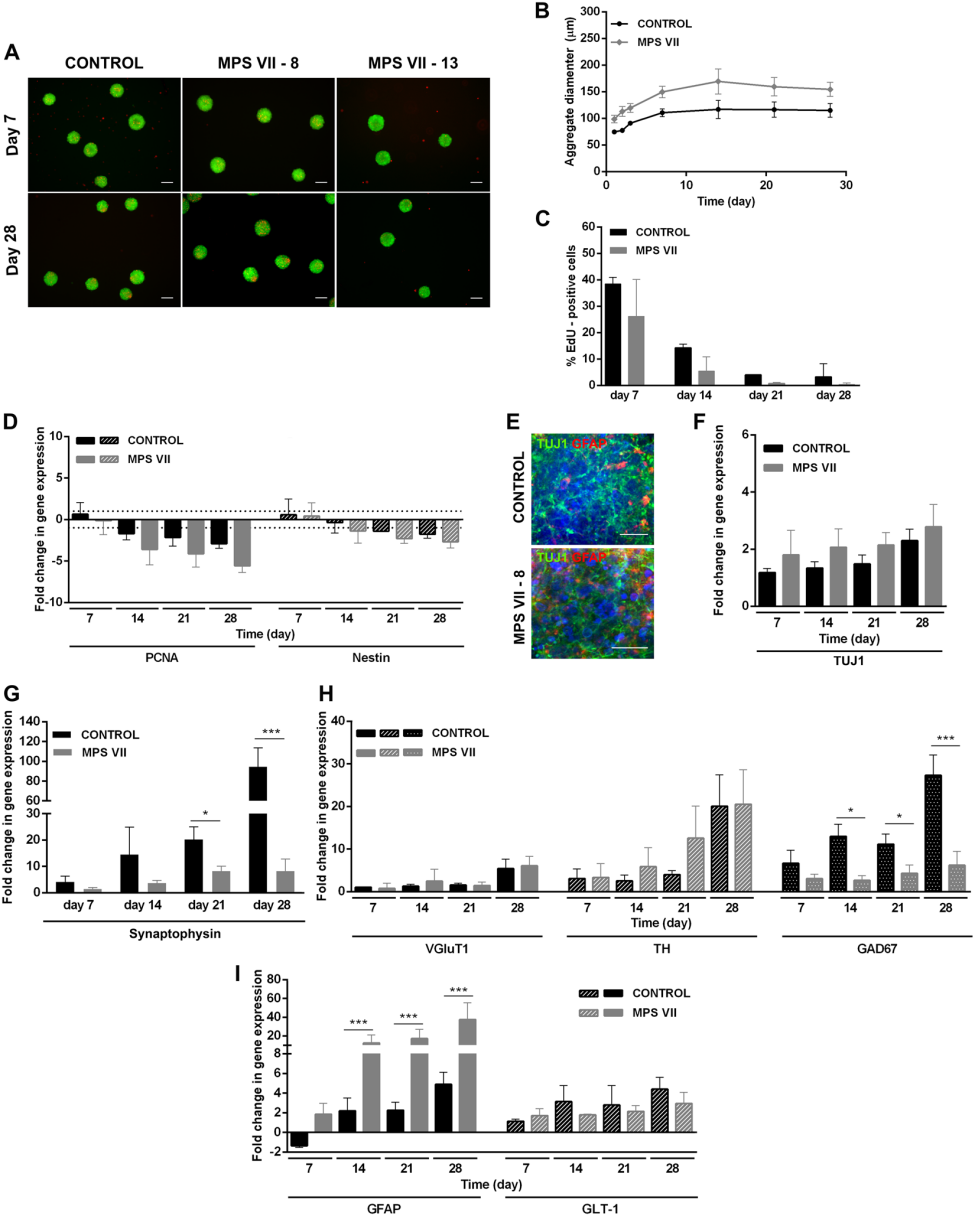
Characterization of MPS VII neurospheroids. (**A**) Representative images of control and MPS VII neurospheroids cell viability at day 7 and 28 by live/dead assay using fluorescein diacetate (FDA) for staining of live cells (green) and propidium iodide (PI) for dead cells (red). (**B**) Control (black) and MPS VII (grey) neurospheroid diameter profile along culture time. (**C**) Percentage of EdU^+^ cells in control (black) and MPS VII (grey) neurospheroids along culture time. (**D**) Gene expression analyses of control (black) and MPS VII (grey) neurospheroids; fold-changes (normalized to undifferentiated cells) of proliferation (*PCNA*) and NPC (*nestin*) mRNAs. Data are mean ± SD of 3 (control) and 4 (MPS VII; 2 with MPS VII-cl.8 and 2 with MPS VII-cl.13) independent cultures. (**E**) Immunofluorescence confocal microscopy of control and MPS VII neurospheroids stained for astrocytic GFAP (red), neuronal βIII-Tubulin (green) and DAPI (blue); scale bars 20 µm. Gene expression analyses of control (black) and MPS VII (grey) neurospheroids; fold-change (normalized to undifferentiated cells) of: (**F**) *βIII-tubulin*, (**G**) the synaptic vesicle marker *synaptophysin*, (**H**) glutamatergic (*VGluT1*), dopaminergic (*TH*) and GABAergic (*GAD67*) neuronal subtypes markers, (**I**) astrocytic markers *GFAP* and *GLT1*. Data are mean ± SD of 3 (control) and 4 (MPS VII; 2 with MPS VII-cl.8 and 2 with MPS VII-cl.13) independent cultures. Asterisks indicate significant difference: *p < 0.05, ***p < 0.001.

For 3D differentiation, NPCs were inoculated as single cells in stirred-tank bioreactors, cultured for 1 week in pro-neural medium to induce cell aggregation, and then cultured in NIM for 3 weeks. With this strategy, the neurospheroids contain a mix of neural cells and allow accumulation of endogenous ECM components^19,20^. Control and MPS VII neurospheroids maintained high cell viability along culture time (Fig. 4A) and spheroid size was stable after the aggregation period (Fig. 4B). NPC proliferation, evaluated by the percentage of EdU^+^ cells, decreased along culture time suggesting differentiation towards a post-mitotic neuronal lineage (Fig. 4C). Concomitantly, downregulation of proliferation (*PCNA*) and NPC (*nestin*) mRNAs was observed along culture time (Fig. 4D), with no differences between control and MPS VII cells. These data are in line with 2D differentiation, indicating that MPS VII NPCs have no apparent alterations in the potential to undergo neural differentiation. Neuronal differentiation in 3D cultures was confirmed by gene expression and detection of βIII-tubulin^+^ cells within neurospheroids by day 28 of culture (Fig. 4E,F). Importantly, gene expression of the pre-synaptic vesicle marker synaptophysin (*SYP*) was significantly lower in MPS VII neurons (Fig. 4G), suggesting that synaptic dysfunction might occur. The upregulation of *vGluT1*, *TH* and *GAD67* mRNAs (Fig. 4H) suggested the presence of glutamatergic, dopaminergic and GABAergic neuronal subtypes, respectively. Moreover, a significantly (p < 0.05) lower (4-fold) level of *GAD67* gene expression was detected in MPS VII neurospheroids at day 28 (Fig. 4H), suggesting decreased numbers of GABAergic inhibitory neurons.

An increase in the astrocytic cells within 2D and MPS VII neurospheroids was consistent with the time-dependent increase in gene expression of *GFAP* and of *GLT-1*, which codes for a glutamate transporter (Fig. 4I). Notably, significant higher *GFAP* gene expression was observed in MPS VII neurospheroids (Fig. 4I), while no differences in *GLT-1* were observed, suggesting that the number of potential astrocytes was not affected. Together, these results suggest that the increase in *GFAP* mRNA was not directly related to astrocyte differentiation efficiency, but possibly related to MPS VII astrocyte activation, which may be a consequence of GAG accumulation and interaction with pattern recognition receptors^23^.

**Figure 5:**
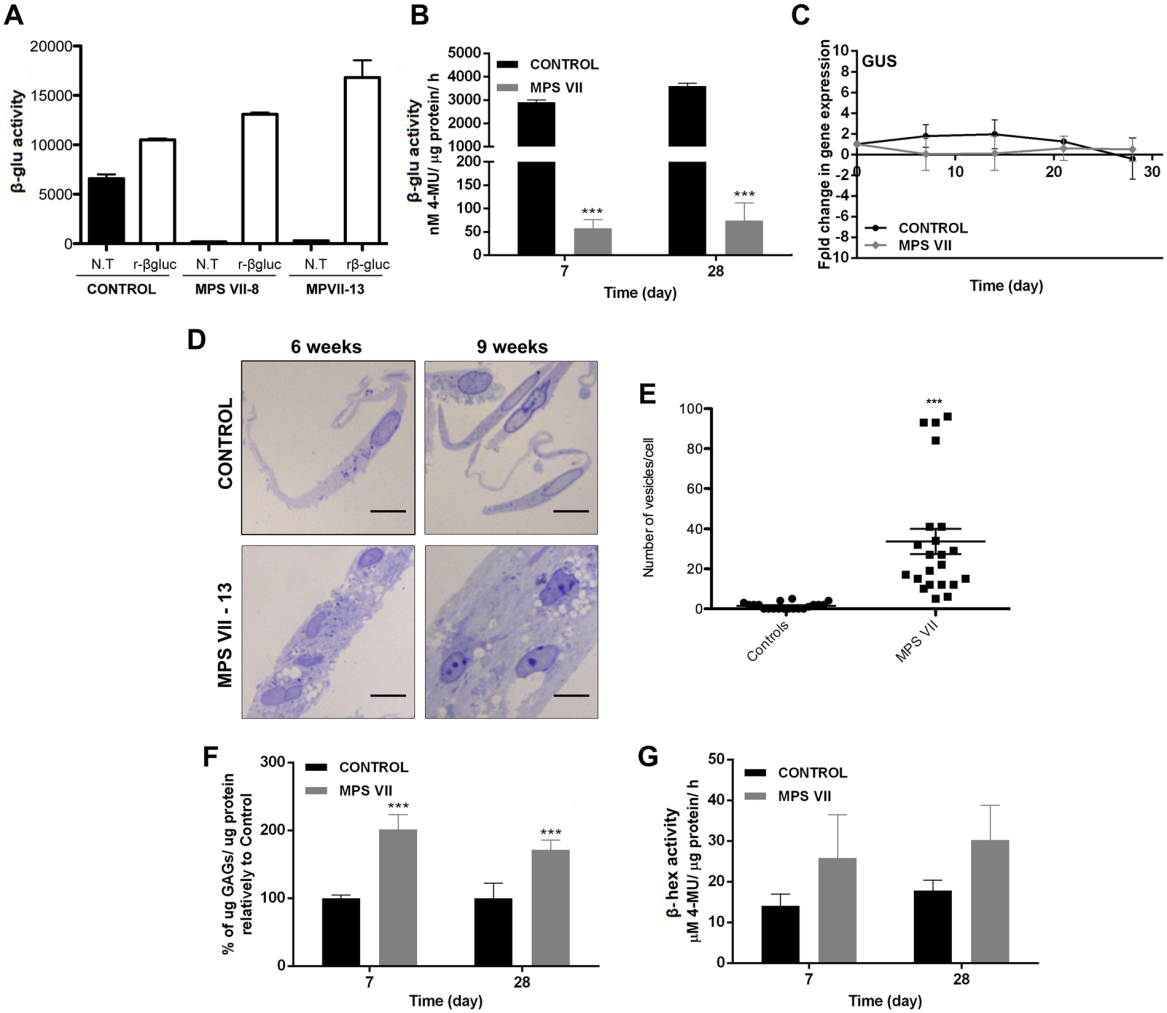
MPS VII neural cells recapitulate known disease features. (**A**) β-gluc enzymatic activity (expressed in nmol 4-MU/μg of protein/h) in control and MPS VII neurons, non-treated (N.T.) and treated with rβ-gluc. (**B**) β-gluc enzymatic activity in control (black) and MPS VII (grey) neurospheroids at day 7 and 28. (**C**) Gene expression analysis of control (black) and MPS VII (grey) neurospheroids; fold-change (normalized to undifferentiated cells) of *GUSB*. (**D**) GAGs storage in control and MPS VII neurons by toluidine blue staining at 6 and 9 weeks; scale bars 5 μm. (**E**) Number of toluidine blue nonstaining vesicles per cell at week 9; micrographs were analyzed using ImageJ software and the number of nonstaining vesicles/cell, with a minimum of 0.5 and a maximum of 4.2 μm, were counted manually by a masked observer; each data point (n) corresponds to a single, well isolated, cell (control n = 20, MPS VII n = 23). (**F**) GAGs quantification in control (black) and MPS VII (grey) neurospheroids by Blyscan Sulphated Glycosaminoglycan assay. (**G**) β-hex enzymatic activity (expressed in nmol 4-MU/μg of protein/h) in control (black) and MPS VII (grey) neurospheroids at day 7 and 28. Data are mean ± SD of 3 (control) and 4 (MPS VII; 2 with MPS VII-cl.8 and 2 with MPS VII-cl.13) independent cultures. ***p < 0.001.

β-gluc activity in extracts of MPS VII neurons (2D) and neurospheroids (3D), was up to 50-fold lower levels compared to control neurons (Fig. 5A,B). After treatment with rβ-gluc, the activity was restored to levels similar to controls (Fig. 5A) suggesting that rβ-gluc reached the lysosomes in MPS VII neurons, as observed for NPCs (Fig. 2B). These results are in line with the low enzymatic activities found in MPS VII patient fibroblasts (Fig. 1A) and iPSC-NPCs (Fig. 2B). In addition, *GUSB* mRNA levels were stable along culture time in control and MPS VII neurospheroids (Fig. 5C). GAG accumulation, secretion and presence of enlarged intracellular vesicles are the main histological hallmarks and biomarkers for MPS VII^2^. We first investigated whether MPS VII neurons accumulate GAGs and harbor extended vesicles. By using toluidine blue staining, we found more and bigger non-stained vesicles in MPS VII neurons versus control neurons (Fig. 5D), which was approximately 20-fold greater at week 9 (Fig. 5E). In MPS VII neurospheroids, total GAG content was approximately two times higher than in of controls, and this increase was already observed by day 7 of culture and maintained up to day 28 (Fig. 5F). This phenotype in human MPS VII neurons resembles the one observed in murine^24,25^ and canine^26^ MPS VII brain. Additionally, in several MPSs one can detect increased expression of other genes coding for lysosomal enzymes under the control of TFEB (transcription factor EB)^27^. β-hexosaminidase (β-hex) is one lysosomal enzyme whose production and activity are elevated when the greater lysosomal system is perturbed^27^. Notably, we observed a 1.7-fold increase in β-hex activity in the MPS VII neurospheroids at days 7 and 28 (Fig. 5G). Similarly, increased β-hex activity had been reported in MPS VII murine and canine brains^24,26^. Together, these data demonstrate that we generated human iPSC-derived MPS VII neurons that harbored the histological hallmarks of MPS VII pathology.

### Lysosomal structural alterations in MPS VII NPCs and neurons

**Figure 6:**
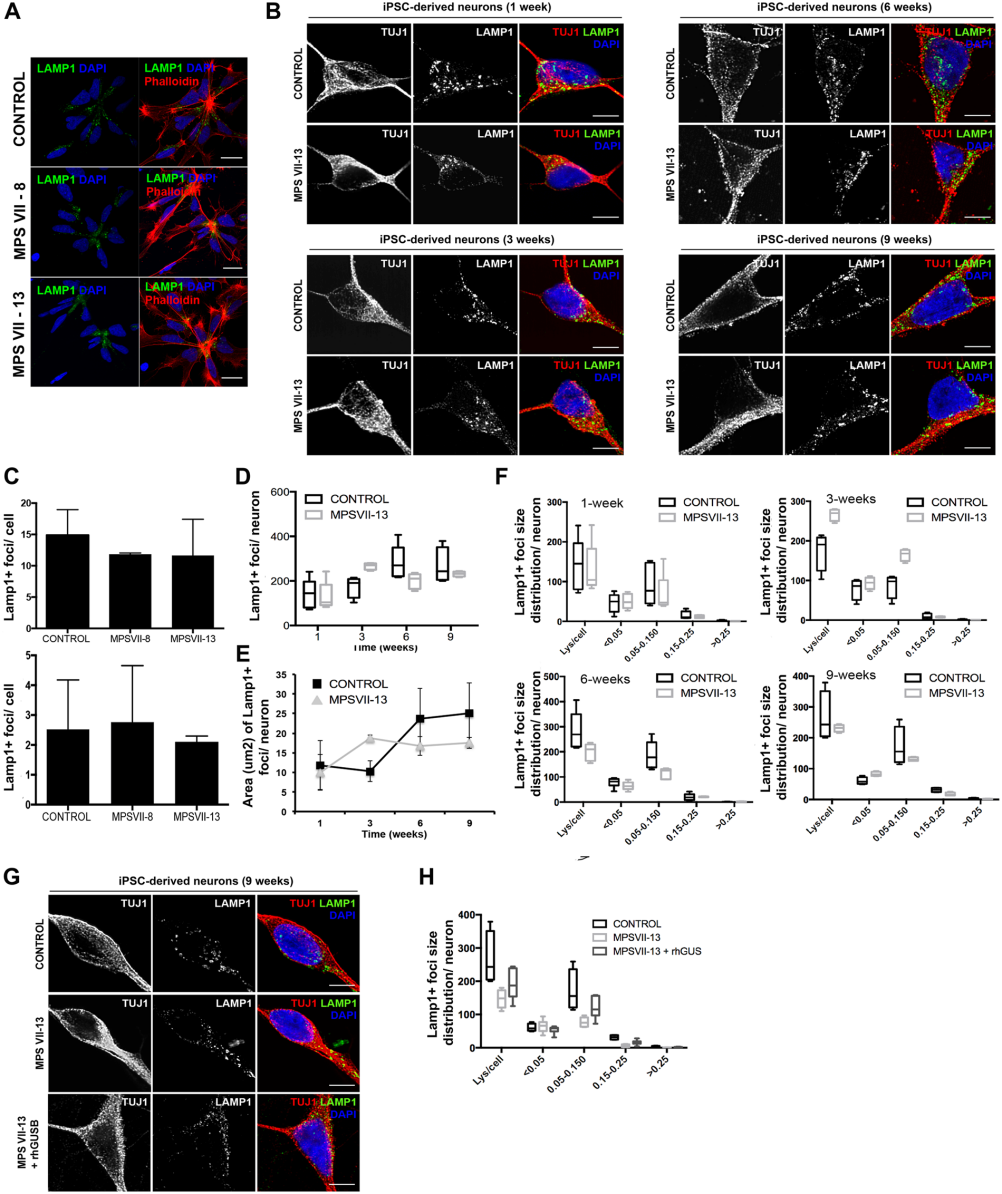
Lysosome structural alteration in MPS VII NPCs and neurons. (**A**) Immunofluorescence microscopy of control and MPS VII NPC, stained for LAMP1 (green) and DAPI (blue; left panel) or phalloidin (red) and DAPI (blue; right panel); scale bars 20 µm. (**B**) Immunofluorescence confocal microscopy of control and MPS VII neurons at 1, 3, 6 and 9 weeks of culture, stained for LAMP1 (green), βIII-tubulin (TUJ1, red) and DAPI (blue); scale bars 5 µm. (**C**) Quantification of acidic vesicles labeled with Lysotracker^TM^ of control and MPS VII NPC, with areas between 0.1–1.2 μm^2^ per cell (top) and higher than 1.2 μm^2^ per cell (bottom). Mean LAMP1^+^ vesicles content (**D**), area (µm^2^) (**E**) and size distribution (**F**) per neuron for control and MPS VII neurons along culture time. (**G**) Immunofluorescence confocal microscopy of control, MPS VII neurons and β-gluc-treated MPS VII neurons at 9 weeks of culture, stained for LAMP1 (green), βIII-tubulin (TUJ1, red) and DAPI (blue); scale bars 5 µm. (**H**) Mean LAMP1^+^ vesicles size distribution for control, MPS VII neurons and β-gluc-treated MPS VII neurons at 9 weeks of culture.

As subcellular distribution and size of lysosomes can be indicative of perturbations in the lysosome system, we quantified these parameters in MPS VII NPCs and neurons using lysosome-associated membrane protein 1 (LAMP1) immunoreactivity as a readout. A caveat in this readout is that while confocal microscopy can readily detect signals as small as 10 nm, it does not allow one to differentiate between large structures and clustered smaller structures. We found that control and MPS VII NPCs have lysosomes located preferentially near the contact point with other NPCs (Fig. 6A). In addition, we found no differences in the number or size of the fluorescent signal of LAMP1 immunoreactivity and acidic vesicles, as measured by Lysotracker, in NPCs (Fig. 6B,C). The number and area LAMP1 immunoreactive foci were also quantified in neurons during 9 weeks of differentiation (Fig. 6B). While in the first week no differences were found in the number or area, between weeks 1 and 3 LAMP1 immunoreactive foci were modestly increased in number and area in MPS VII neurons compared to control. However, these differences decreased in weeks 6 and 9 where the number and area of lysosomal vesicles were slightly lower in MPS VII neurons (Fig. 6D,E). Therefore, we quantified LAMP1 immunoreactive foci size distribution at each time point. Similar to that observed for lysosome area, at week 1 no difference was observed between control and MPS VII neurons (Fig. 6F). However, by week 3 we found variations in foci sizes ranging between 0.05–0.150 μm^2^ in MPS VII neurons compared to control neurons (Fig. 6F). Notably, there were no differences in LAMP1 immunoreactive foci smaller than 0.05 μm^2^ or bigger than 0.150 μm^2^, suggesting that the variations in the number and area were not due to an accumulation of LAMP1^+^ vesicles. Together, these data suggest that in MPS VII neurons, LAMP1^+^ vesicles size differs from control neurons. To determine if ERT can correct this phenotype, 3 week-old MPS VII neurons were incubated with rβ-gluc for 3 weeks and the number, size, and distribution of LAMP1 immunoreactive foci were characterized. We found that ERT partial restored LAMP1 immunoreactive patterns with foci ranging between 0.05–0.150 μm^2^ slightly increased in β-gluc-treated MPS VII neurons compared with mock-treated MPS VII neurons (Fig. 6G,H). These data indicate that the variation in LAMP1^+^ immunoreactivity in MPS VII neurons can be partially restored to that of healthy cells by ERT.

### TEM reveals anomalies in endocytic compartments of MPS VII neurons

**Figure 7:**
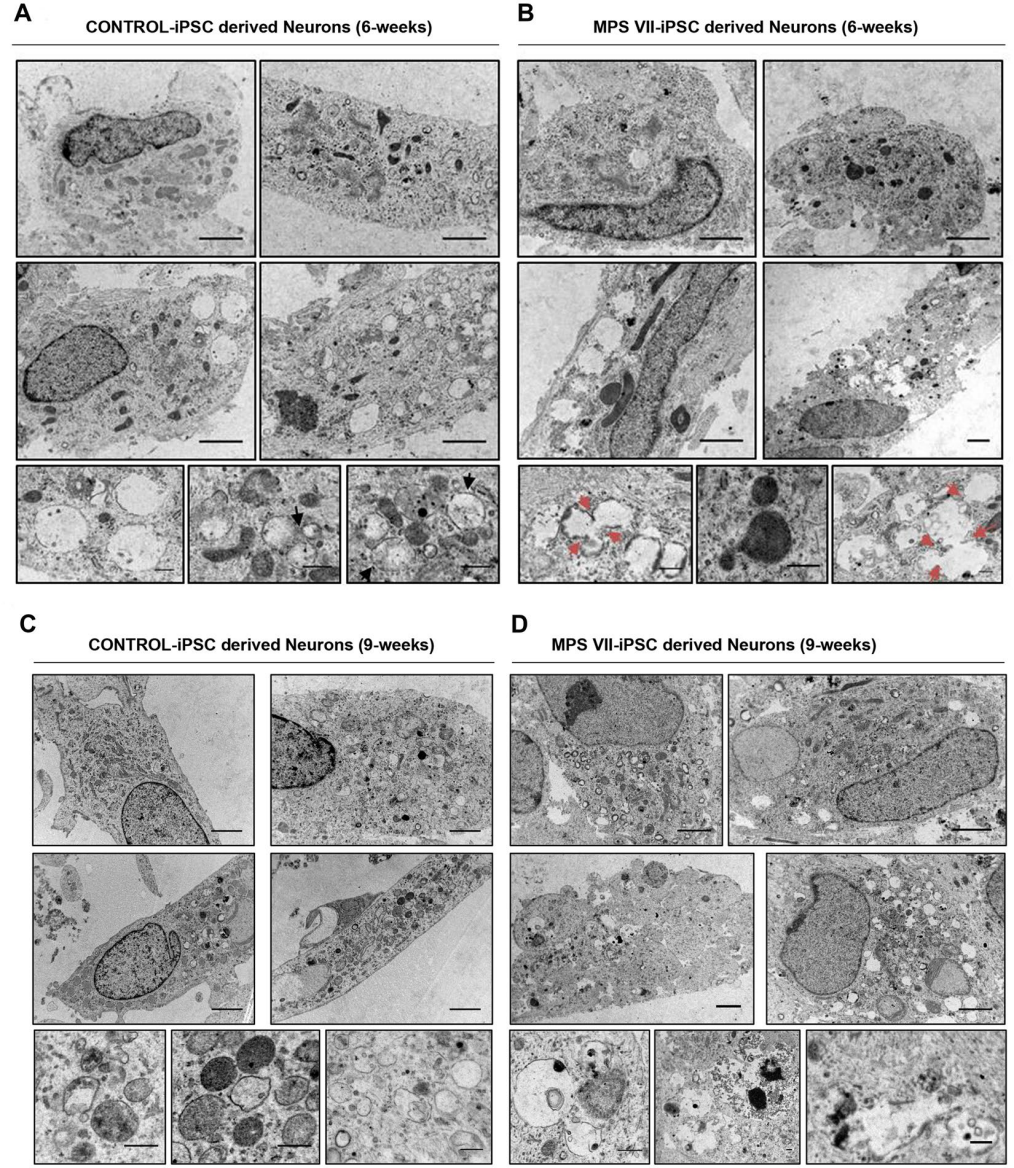
Transmission electron microscopy (TEM) of MPS VII neurons. Ultrastructure of control (**A**,**C**) and MPS VII (**B**,**D**) neurons at 6 (**A**,**B**) and 9 (**C**,**D**) weeks of culture. Black arrows in (**A**) show well-defined endocytic compartment with amphisomes and multivesicular bodies in control neurons, while MPS VII neurons harbor expanded and heterogeneous endocytic compartments that formed clusters. Red arrows in (**B**) indicate fusion between homotypic vesicles in MPS VII neurons. Scale bars 2 μm (top panels) and 0.5 μm (bottom panels).

Transmission electron microscopy (TEM) was used to examine differences in the endocytic compartment between control and MPS VII neurons at 6 and 9 weeks of culture. In culture conditions that mimic the suboptimal conditions in the MPS VII brain, control neurons contained more double membrane structures that MPS VII neurons (Fig. 7). Consistent with the immunofluorescence studies, control neurons contained a well-defined endocytic compartment with amphisomes and multivesicular bodies (Fig. 7A,C), while MPS VII neurons harbored expanded endocytic compartments that were heterogeneous and formed clusters (Fig. 7B,D). TEM confirmed the presence of lipofuscin granules and micro-vacuolation of neuronal cell cytoplasm with fusion between homotypic vesicles, suggesting defects in membrane fusion and compromised neuronal lysosomal function (Fig. 7A,D). These data identify pathological changes in human MPS VII neurons that are suggestive of higher autophagic activity, which may impact learning and memory disabilities observed in MPS VII mice and some MPS VII patients.

### Functional alterations in the greater lysosomal system of MPS VII neurons

**Figure 8:**
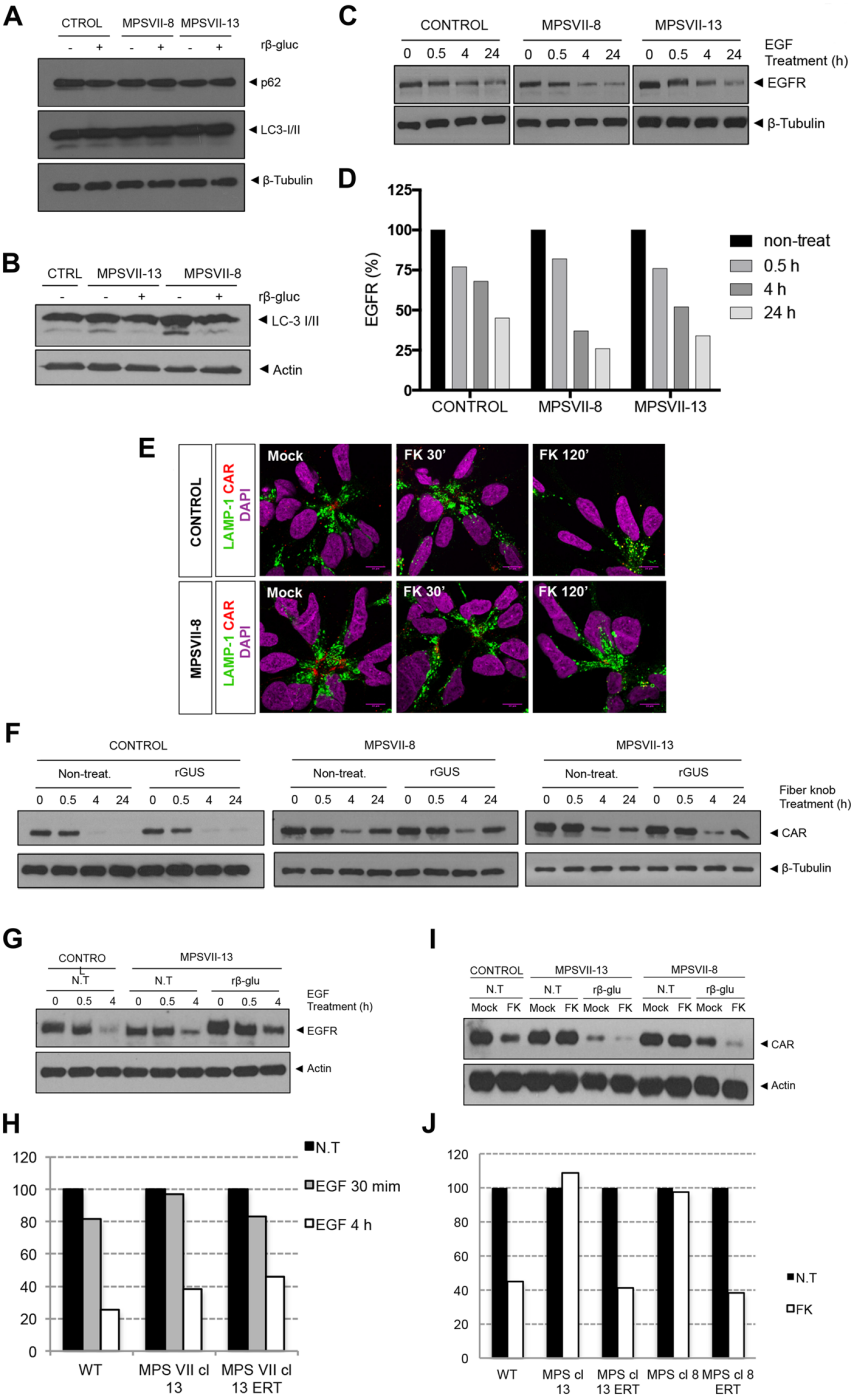
Lysosome function alteration in MPS VII NPCs and neurons. Autophagy basal levels in control and MPS VII NPCs (**A**) and in control and MPS VII neurons (**B**). P62 and LC3-I/ II were analyzed by western blot of control and MPS VII NPC protein extracts, non-treated (−) and treated (+) with rβ-gluc. Data from one representative experiment of 4 independent experiments; Images from different parts of the same gel. (**C**) Control and MPS VII NPC lysosome ability to degrade membrane proteins internalized by endosomes assayed by an EGFR degradation assay. EGFR content analyzed by western blot. Data from one representative experiment of 2 independent experiments; Image from different parts of the same gel. (**D**) Quantification of EGFR degradation efficiency in control and MPS VII NPC normalized to β-tubulin levels. (**E**) Immunofluorescence confocal microscopy of control and MPS VII NPC, after induction of CAR degradation, stained for LAMP1 (green), CAR (extra- and intracellular domains, red) and DAPI (blue). CAR localized at the membrane, internalized into lysosomes (LAMP1 and CAR co-localization, 30′) and degraded (120′). (**F**) Lysosome ability to degrade CAR assayed in control and MPS VII NPC, non-treated (non-treat.) and treated with rβ-gluc. CAR content analyzed by Western blot. Data from one representative experiment of 2 independent experiments; Control, MPS VII-8 and MPS VII-13 gels are independent and CAR and β-tubulin images are from different parts of the same gel. (**G**) Lysosome ability to degrade membrane proteins internalized by endosomes assayed by an EGFR degradation assay in control and MPS VII neurons, non-treated (N.T.) and treated with rβ-gluc. EGFR content analyzed by Western blot. Data from one representative experiment of 5 independent experiments; Image from different parts of the same gel. (**H**) Quantification of EGFR degradation efficiency in control and MPS VII neurons normalized to actin levels. (**I**) Control and MPS VII neurons lysosome ability to degrade CAR, non-treated (N.T.) and treated with rβ-gluc. CAR content was analyzed by Western blot. Data from one representative experiment of 5 independent experiments; Image from different parts of the same gel. (**J**) Quantification of CAR degradation efficiency in control and MPS VII neurons normalized to actin levels.

We next asked if other aspects of the greater lysosomal system were altered in MPS VII NPCs and neurons. Lysosomes fuse with autophagosomes to degrade long-lived proteins, organelles and pathogens. The autophagosome marker, LC3-I/II and the adaptor molecule p62/SQSTM1, which label molecules to be delivered to autophagosomes, were used to probe steady-state autophagy levels in NPCs and differentiated neurons. The amount of LC3-I/II and p62/SQSTM1 were similar in control and MPS VII NPCs ± rβ-gluc (Fig. 8A), suggesting that autophagy was not unbalanced in MPS VII NPCs. By contrast while cytosolic LC3-I levels were similar in MPS VII and control neurons, LC3-II basal levels were increased in MPS VII neurons compared to controls, and restored to control levels when rβ-gluc was added to the medium (Fig. 8B). These results suggest a defect in autophagosome-lysosome fusion (autophagosome maturation) or a dysfunction of lysosomal protein degradation in MPS VII neurons.

In addition to intracellular degradation of host proteins, lysosomes also play a role in the regulation of signaling pathways by degrading cell surface receptors. Epidermal growth factor (EGF) signal transduction requires EGF receptor (EGFR) engagement, internalization of the EGF-EGFR complex into endosomes and degradation of EGFR in lysosomes, while EGF signaling endosomes reach the nucleus. We therefore addressed the function of endo-lysosomal system by determining if EGFR is efficiently degraded in MPS VII NPCs and neurons. EGFR levels in cell extracts at basal levels and 0.5, 4 or 24 h after recombinant EGF treatment were analyzed by immunoblotting. We found that EGFR levels decreased over time in control and MPS VII NPC, with a 60–75% loss at 24 h, indicating that lysosomes functioned properly for EGFR degradation (Fig. 8C,D). To address lysosomal function using another cell surface protein, receptor-mediated endocytosis and lysosomal degradation was assayed for coxsackievirus and adenovirus receptor (CAR)^28^. We previously showed that the canine adenovirus type 2 fiber knob (FK^CAV^) binds CAR^29^ and induces its lysosome-mediated degradation in neuronal cells^30^. Importantly, EGFR and CAR occupy different microdomains in the plasma membrane: EGFR is globally spread while CAR is enriched in lipid rafts^30^, and therefore different internalization routes are used. MPS VII NPCs were incubated with FK^CAV^ and CAR internalization and degradation were followed by CAR and LAMP1 immunoreactivity. In resting conditions, CAR was preferentially found at the cell membrane that contacts other NPCs. Thirty minutes post-incubation with FK^CAV^, CAR was internalized and co-localized with LAMP1 **(**Fig. 8E**)**. At 2 h, CAR levels decreased compared with resting conditions, indicating its degradation both in control and MPS VII NPCs **(**Fig. 8E). CAR levels were also quantified by immunoblotting (Fig. 8F). Control and MPS VII NPCs ± rβ-gluc degraded CAR to a comparable extent (Fig. 8F). These data suggest that lysosome mediated degradation by MPS VII NPCs is not notably altered and that lysosomes function properly in terms of autophagy and receptor degradation.

Because neurons use specific signaling pathways related to membrane remodeling, axonal growth and synapses plasticity, these pathways could be more susceptible to metabolite accumulation and perturbations of the endo-lysosomal system. Thus, levels and processing of EGFR and CAR were also evaluated in MPS VII neurons. We found that the levels of EGFR after EGF treatment were reduced in control, MPS VII and rβ-gluc treated MPS VII neurons (Fig. 8G,H). Moreover, ERT had no effect in EGFR levels, further indicating that there were no alterations in EGFR degradation due to MPS VII pathology. By contrast, CAR levels diminished to 40% after FK^CAV^ treatment in control neurons (Fig. 8I,J), while in MPS VII neurons were unchanged, indicating an alteration in the endo-lysosomal pathway. Of note, CAR levels were diminished in rβ-gluc-treated MPS VII neurons similar to control neurons (Fig. 8I,J). These data are consistent with specific defects in the greater lysosome system of MPS VII neurons.

### Network alterations in MPS VII neurons

**Figure 9:**
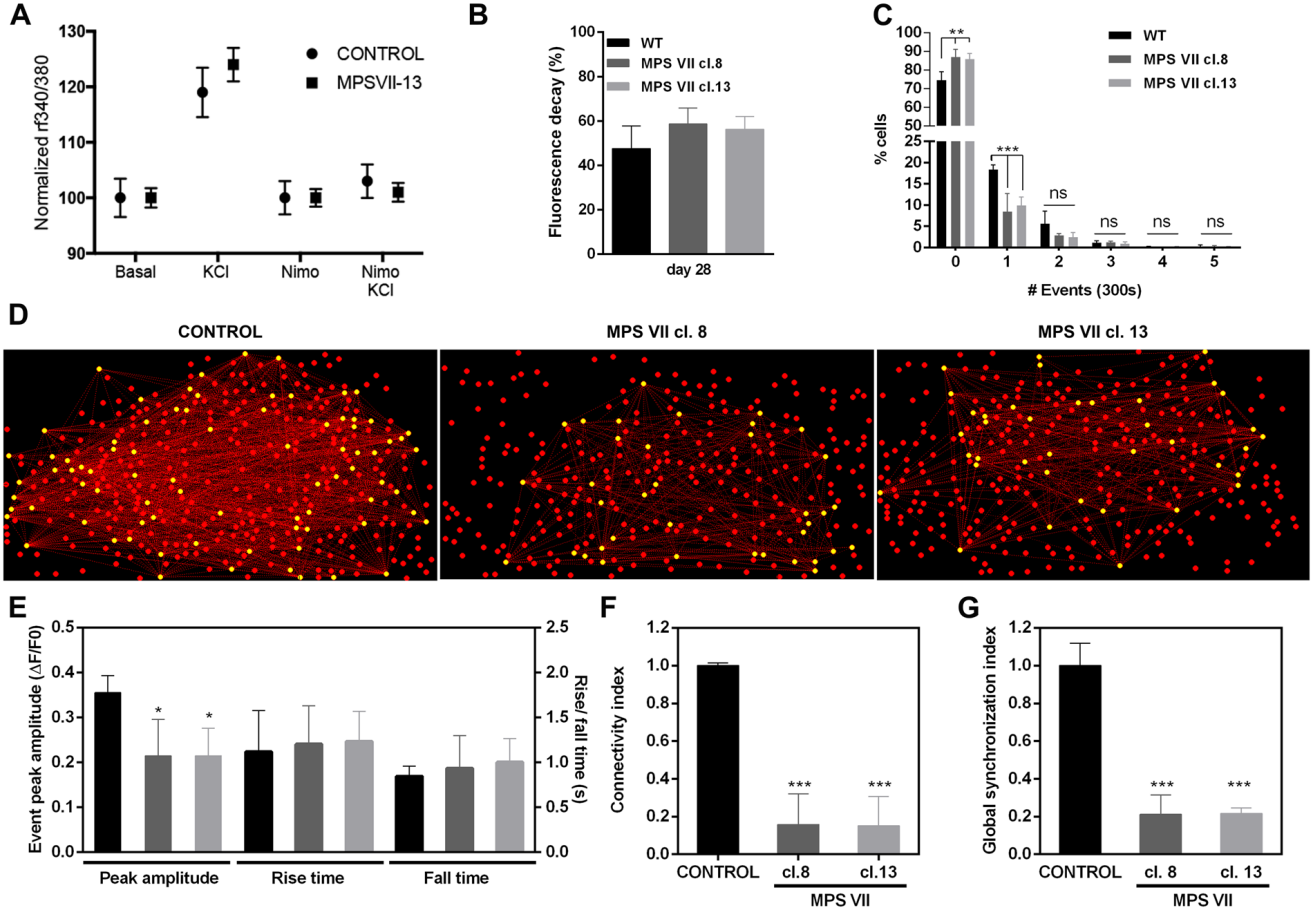
MPS VII neuronal activity and MPS VII neurospheroids calcium (Ca^++^) imaging analysis. (**A**) Ca^++^ release in control and MPS VII neurons, upon KCl induction and reduction of Ca^++^ signaling upon inhibition with nimodipine. (**B**) FM-1–43 fluorescence decay in control (black) and MPS VII (grey) neurospheroids at day 28. Data are mean ± SD of 3 independent cultures. (**C**) Percentage of cells per number of spontaneous Ca^++^ events in 300 seconds for control (black) and MPS VII (cl. 8 grey; cl. 13 light grey) neurospheroids. (**D**) Visual representations of neural networks from control and MPS VII neurospheroids reconstructed using FluoroSNNAP; circles show the position of cells in culture, yellow circles represent functionally connected nodes and red lines represent the functional connectivity of pair-wise neurons. (**E**) Ca^++^ events peak amplitude, rise time and fall time in control (black) and MPS VII (cl. 8 grey; cl. 13 light grey) neurospheroids. Network properties: Connectivity index (**F**) and Global synchronization index (**G**) in control (black) and MPS VII (cl. 8 grey; cl. 13 light grey) neurospheroids. Data are mean ± SD of 3 (control) and 4 (MPS VII; 2 with MPS VII-cl.8 and 2 with MPS VII-cl.13) independent cultures. Asterisks indicate significant difference: *p < 0.05, ***p < 0.01, ***p < 0.001.

MPS VII neuron functionality was assessed by imaging cytosolic calcium (Ca^++^) concentration using the ratiometric fluorescent dye Fura-2, which monitors Ca^++^ entry through voltage-gated Ca^++^ channels in neurons. Upon KCl-induced depolarization, approximately 50% of the cells exhibited signals in both control and MPS VII cultures (Fig. 9A). The effectiveness of nimodipine, a L-type Ca^++^ channel blocker, to inhibit Ca^++^ signals in depolarizing conditions further demonstrated the presence of differentiated neurons in both conditions (Fig. 9A). These data indicated that after 7 weeks in culture MPS VII neurons were functionally similar to control neurons. In MPS VII neurospheroids synapse function was initially assessed by imaging KCl-induced synaptic vesicle exocytosis. Control and MPS VII neurospheroids responded similarly to the depolarizing stimuli, indicating no differences in total synaptic vesicle trafficking (Fig. 9B). Next, Ca^++^ fluorescence imaging was used to evaluate the differences in spontaneous neuronal activity between control and MPS VII neurons and alterations in neuronal network functional connectivity within MPS VII neurospheroids. MPS VII neurospheroids had significantly lower neuronal activity than control, with 8.5% (MPS VII cl.8) and 10.0% (MPS VII cl.13) of cells presenting one spontaneous Ca^++^ event, compared to 18.4% in control neurospheroids, and with a higher percentage of cells that did not present spontaneous Ca^++^ events during the monitoring time (Fig. 9C).

To characterize MPS VII neuronal networks, connectivity was evaluated using FluoroSNNAP software, which allows one to infer functional connectivity between neurons using the temporal correspondence of Ca^++^ events. These analyses generate maps of neuronal interactions where one can quantify the connectivity indexes for each active cell^31^. Representative connectivity maps of control and MPS VII neurospheroids are provided in Fig. 9D. The average peak amplitude of the spontaneous Ca^++^ events of MPS VII neurospheroids was significantly (p < 0.05) smaller than control neurospheroids, while no significant differences were found in rise and fall times (Fig. 9E).

In control network structures, most of the cells established several connections with other cells (Fig. 9D). By contrast, connections were formed only between some of the cells in MPS VII network, leaving the remaining cells weakly connected or disconnected (Fig. 9D). Concomitantly, the average connectivity index for active cells was significantly (p < 0.001) lower in MPS VII neurospheroids (Fig. 9F). A similar pattern was observed for the global synchronization index, which was also significantly (p < 0.001) lower in MPS VII networks (Fig. 9G), further indicating alterations in MPS VII neuronal networks. Together, these results demonstrate that MPS VII neurospheroids have less active cells with smaller Ca^++^ peak amplitudes than heathy control neurospheroids. This leads to cells that are weakly connected and poorly synchronized, which may impact the MPS VII brain network topology and functionality.

## Discussion

One of our primary goals at the core of our studies is to identify quantifiable neuronal phenotypes that could complement genetic analyses to help predict the severity of the heterogeneous MPS VII-associated cognitive anomalies. The range of pathological MPS VII features makes prognosis of disease progression, and in turn recommending treatment options, challenging. Several years ago we reasoned that the logarithmic pace of technological improvements in cellular reprograming and directed differentiation will allow one to rapidly and inexpensively generate neural cells from many cell types. Transdifferentiation of human peripheral blood T cells into neurons has now been reported^32^ and the unique functions of neural cells can be probed for MPS VII-associated defects in a timely and cost-effective manner.

Here, we used human MPS VII iPSC-derived neural cells, cultured in 2D and 3D culture systems, to try to identify neuronal defects. We found that ERT, in this case supraphysiological levels of β-gluc activity, was not necessary for fibroblast reprogramming, iPSC generation or expansion. The requirement for ERT is similar to that described for MPS IIIC iPSC^13^, but in contrast to that reported for MPS IIIB and Pompe disease iPSC^11,12^. Generating NPCs is primordial during normal brain development. Despite limited β-gluc activity, a homogeneous, expandable and phenotypically stable population of NSCs was obtained. These *in vitro* data are consistent with the clinical manifestation of MPS VII, and with early brain development of MPS VII mice and dogs, which appears histologically normal^24–26^. These data suggest that deficiency in β-gluc activity may not have a discernable impact on initial stages of neural development. A caveat is that during fetal development maternal β-gluc would be present for uptake in brain cells until the fetal blood brain barrier is formed.

We then asked whether some aspects of neural cell homeostasis could be perturbed by decreased β-gluc activity. We show that MPS VII neurons differentiated in 2D cultures formed functional synapses. Similar differentiation patterns were observed during 2D differentiation of control and MPS VII neurons indicating that reduced β-gluc activity did not affect the onset of the neuronal differentiation of MPS VII NPCs in this culture system. Differentiation of MPS VII NPCs in a 3D culture system revealed downregulation of proliferating cells, prototypic NPC gene expression, and upregulation of neuronal βIII-tubulin, consistent with the ability of MPS VII NPCs to undergo neuronal differentiation. Neuronal differentiation was consistent with the expression of synaptophysin and markers of glutamatergic, dopaminergic and GABAergic neurons. Notably, *GAD67* gene expression was lower in MPS VII neurospheroids, suggesting that there was a reduction in GABAergic neuronal differentiation. GABA is the main inhibitory neurotransmitter within the brain and GABAergic neurons are involved in several physiological functions, including stabilization of neuronal activity. A balance between excitation and inhibition ensures the normal functioning of the neuronal networks^33^, so decreased GABAergic neurons could potentially lead to neuronal networks alterations. Indeed, GABAergic neurons exhibit neuroaxonal dystrophy more than other cells types in a MPS VII mouse model and in other LSD, such as MPS I, and GM1 and GM2 gangliosidosis^34–36^. Concomitantly, a 8-fold down-regulation in OTX1–2 mRNA, which encodes a transcription factor that controls neuron subtype identity and the fate of GABAergic neurons, was found in the brain of a MPS VII mouse^36^ and in the human MPS IIB brain the density of GABAergic neurons in the cerebral cortex was markedly reduced when compared with age-matched controls^37^.

Despite decreased synaptophysin gene expression in MPS VII neurospheroids, functional synapses were equivalent to controls. However, it is possible that this approach, and/or our culture conditions were not optimized to detect subtle differences in neuronal functionality during the depolarization assays. More specifically, overall fluorescence of the neurospheroids was quantified in medium optimized for culturing and maintaining healthy neuronal cells, and therefore this environment poorly recapitulate the environment in the MPS VII brain. Detecting alterations in GABAergic neurons synaptic activity may require a “stressed/challenging” environment. For example, astrocytes provide neurotrophic support, help synchronize neurotransmitter metabolism and release and regulate the extracellular milieu. Thus, alterations in astrocyte function can be a major contributor to neurodegeneration and/or cognitive defects^38,39^. Notably, higher *GFAP* mRNA levels were found in MPS VII neurospheroids. Similarly, in recent studies performing transcriptome analyses of the MPS VII mouse brain, thirteen astrocyte-specific mRNAs (including *GFAP)* were upregulated^36,40^. Upregulation of *GFAP* is the hallmark of mature astrocyte reactivity that occurs in response to an insult or injury in the CNS^38,41^, which can include chronic neurological conditions such as Alzheimer’s disease, Parkinson’s disease or LSDs^42^. Microglial and astrocyte activation are hallmarks of many LSDs that affect the CNS and can promote neurodegeneration by creating a neurotoxic environment due to elevated levels of cytokines, chemokines, and pro-apoptotic molecules. Astrocyte activation has been suggested to impact MPS pathogenesis^43^. Indeed, an increase in the number of GFAP^+^ astrocytes was found in the cerebral cortex of MPS II and IIIB patients^37^. However, the cellular basis and mechanisms responsible for eliciting astrocyte reactivity and neuroinflammatory pathways and its consequences in neural function are not clearly understood^42,44^. Mediators of innate immunity, such as lipopolysaccharide (LPS) and other toll-like receptor (TLR) ligands, as well as neurodegenerative diseases, are able to trigger reactive astrogliosis to different extents^38,39^. In LSDs, intrinsic changes resulting from the accumulation of lysosomal material could be the trigger for astrocyte activation. Of note, GAG breakdown products are structurally similar to LPS, the canonical ligand of TLR4^45,46^. Therefore, GAGs accumulation could activate TLR4 signaling pathway inducing a pro-inflammatory response, eliciting cytokine and reactive oxygen species production. Priming of microglia via the TLR4 pathway has been demonstrated in the mouse MPS IIIC brain^47^ and MPS VII mice presented elevated levels of p-STAT1-Ser727 and p-STAT3-Tyr705, consistent with TLR4 activation^48^. Besides, many LSDs are also associated with defects in autophagy, which could represent another pathway to induce astrocytosis, since cells are unable to clear cellular debris^49^. Further exploring the astrocytes in 3D MPS VII neurospheroids could help us to identify the mechanisms of astrocyte reactivity and neuroinflammation.

While MPS VII NPCs did not show major lysosome structural alterations, MPS VII neurons transiently contained more and larger LAMP1 immunoreactive foci. We can only speculate how the lack of β-gluc activity could induce this phenotype. Lysosome and lysosome related organelle (LRO) biogenesis, size, and turnover are linked to stress and the metabolic state of the cells^50^. However, biogenesis, which includes reformation of lysosomes from endolysosomes and from tubular structures projecting from autolysosomes, is poorly understood even in healthy non-neuronal cells. In addition, lysosome near the plasma membrane can undergo secretion. Transport of LRO in projections is also a feature unique to differentiating and mature neurons. The LRO gradually decrease their pH as they migrate towards the soma prior to fusing with other lysosomes. Each of these pathways may be affected during maturation of MPS VII neurons^51^. In contrast to other neurodegenerative diseases like Alzheimer’s disease^52^, we saw no striking differences in LAMP1 immunoreactivity in neuronal projections. It is possible that the early increase of lysosomal vesicles may be a compensation for an imbalance in early lysosome biogenesis, reduced recycling, a defect in autophagosome-lysosome fusion, and/or a dysfunction of lysosomal protease degradation. In addition to intracellular degradation of host proteins, lysosomes also play a role in the regulation of signaling pathways by degrading cell surface protein receptors. Our data suggested that lysosomes function properly in terms of EGFR degradation both in MPS VII NPCs and neurons and CAR degradation in MPS VII NPC, but not in MPS VII neurons. As neurons use specific signaling pathways related to membrane remodeling, axonal growth and synapse plasticity, these could be more susceptible to metabolite accumulation and perturbations of the endo-lysosomal system. These data suggest that signaling pathways following similar internalization routes like CAR, which is lipid microdomain-, actin-, and dynamin-dependent^30^, may be affected in MPS VII neurons. Importantly, these alterations in MPS VII neurons were restored by ERT.

The functional and networking alterations in the MPS VII neurons are ideal starting points to understand MPS VII cognitive defects. A consequence of lower connectivity and poor synchronization could be related with the decrease in GABAergic neurons. There is a strong interplay between Ca^++^ levels and GABA, as a GABAergic neuron decline is initiated by abnormal increased intracellular Ca^++^ levels^33^. Moreover, as the balance between excitation and inhibition ensures the normal functioning of the networks, a decrease in GABAergic neurons could negatively impact the networks^33^. This disturbance in MPS VII neuronal activity and network functional connectivity is, to the best of our knowledge, the first report of *in vitro* functional impairment that may recapitulate the mechanisms underlying brain dysfunction and cognitive impairment in MPS VII patients. The deregulation of Ca^++^ levels and GABA levels further link LSD and Parkinson’s disease, which shared genetic, clinical and neuropathological features^53^. Furthermore, these observations are important to provide new insights in the relation between alterations in the network in MPS VII disease and GAG’s accumulation and its impact on cognition. Now that quantifiable readouts have been identified, future studies are needed to include iPSC-derived neurons from a cohort of MPS VII patients to identify inter-individual differences to be correlated with patient’s mutations and to provide mechanistic insights on whether disease phenotype affects neural differentiation and neuronal network functionality. This could represent a step towards improved post-natal diagnosis and therapeutic strategies to prevent/reverse MPS VII-associated cognitive defects. Given the similarities between MPS VII and other LSD, our study has broad implications.

In summary, we identified MPS VII-associated perturbations through differentiation of iPSC into NPCs and neurons. Our study provides an iPSC-based model that reproduces the major biochemical and histological features of the MPS VII, especially neuronal phenotypes. This provides a platform upon which the cellular processes responsible for brain dysfunction in MPS VII can be further elucidated and used for testing and optimizing therapies. 3D neurospheroid culture systems combined with neuronal connectivity assays have the potential to assess neurological defects in other LSD and neurodegenerative diseases with variable phenotypes.

## Materials and Methods

### iPSC generation

The skin biopsy was performed at the Vall d’Hebron Hospital. The Vall d’Hebron Hospital ethical committee of clinical investigation approved procedures according to local guidelines and regulations. Informed written consent was obtained from the subject’s mother. All experimental protocols at IGMM were approved by the National Agency for Health and Medicine under the biomedical research authorisation number IGMM-6486. A skin punch biopsy (2 mm^3^) under local anesthesia was taken from MPS VII donor, biological samples were free of pathogens. Skin biopsies were cultured in AmnioMax^TM^ basal medium containing AmnioMax^TM^ C-100 Supplement (Invitrogen), 10% fetal bovine serum (FBS) (Gibco) and a mixture of penicillin-streptomycin-Amphotericin B (Lonza). Dermal fibroblasts that emerged from skin biopsies were expanded 12 days later. Then medium was switched to fibroblasts medium (DMEM-GlutaMAX, 10% FBS, sodium pyruvate, MEM non-essential amino acids and antibiotics (Gibco®)). For reprogramming, fibroblasts were treated with FGF at 10 ng/mL and ascorbic acid 2-phosphate at 1 mM (Sigma) for 12 days prior to infection. Ecotropic retroviral vectors expressing 4 transcription factors (OCT4, cMYC, SOX2 and KLF4) described by Yamanaka^7^. Vectors were produced in Plat-E cells^54^ and used to infect fibroblasts pre-treated with VSV-G vesicles containing mCAT1 receptor to improve human cell infection with murine virus^55^. One week later, infected-fibroblasts were expanded and fibroblast medium was switched to human embryonic stem (ES) cells medium (HES; KnockOut^TM^ DMEM, 20% KnockOut^TM^ Serum Replacement, MEM non-essential amino acids, GlutaMAX^TM^, 2-mercaptoethanol, Gibco). HES medium was supplemented with 500 μm valproic acid sodium salt (Sigma) and 10 ng/ml FGF2 (PeproTech) for 3 weeks to increase reprogramming efficiency and maintain cells undifferentiated^56^. A total of 20 ES-like colonies were picked for each specimen 20–35 days later. BJ1-FGF2 (provided by I-Stem) and HFF1 (ATCC) cells were used as feeders. iPSC were mechanically passaged weekly, and medium was changed daily. Rock inhibitor Y-27632 at 10 μm (Calbiochem) was added to the medium at the first passages to improve single cell survival and cell attachment to feeder cells. Three iPSC lines were generated from the MPS VII patient and seven iPSC lines from a control were used. Two MPS VII clones (cl. 8 and cl. 13) were further characterized. Mycoplasma test (MycoAlert® Detection Kit, Lonza) was routinely performed.

### Characterization of iPSC lines

Alkaline phosphatase (AP) activity was detected by a colorimetric assay following manufacture indications (SIGMA FAST™ BCIP/NBT tablets). Expression of pluripotent cell surface markers (SSEA-3, TRA-2–49 and NANOG) were assayed in iPSCs grown on HFF1 feeders layer by immunohistochemical assays (see below). RT-qPCR was done as previously described^57^. Gene expression was normalized to that of glyceraldehyde 3-phosphate dehydrogenase (*GAPDH*).

*In vitro* differentiation towards the three germ layers was performed by embryoid body formation. Briefly, iPSCs were detached from feeder cells with collagenase type IV at 1 mg/ml (Invitrogen) and cells were grown in suspension for 7 days in HES media. For further differentiation, cells were plated into 0.1% gelatin-coated plates (Sigma). Then, 14 days later, cells were fixed with 4% paraformaldehyde (PFA) (EM grade, PKS) in PBS for 15 min and expression of tissue specific markers for endoderm (α-fetoprotein), mesoderm (smooth muscle actin), and ectoderm (TUJ1) were assayed by immunofluorescence (see below).

For teratoma formation, CB17/lcr-Prkdcscid/lcrlcoCrl Fox Chase SCID Congenic mice (Charles River Laboratories) were used. Eight-week old mice were anesthetized with xylazine (5–10 mg/kg): ketamine (100 mg/kg) mix. Cell injections were intratesticular and under the kidney capsule^58–60^ with 10^6^ cells resuspended into 10 μl of DMEM/F12 (Gibco) and 10 μl of Matrigel (BD)^59^. Eight weeks after injection, tumors were removed and fixed in 4% neutral buffered formalin (Diapath) at room temperature (RT) for 24 to 48 h. Samples were processed, included in paraffin and stained with hematoxylin/eosin at the RHEM histology platform (IRCM, Montpellier). Histological analysis was performed by a pathologist. The protocol followed for teratoma formation takes into account standardization rules^61^. All animal experiments were conducted following protocols previously approved by the Comité National de Réflexion Ethique sur l′Expérimentation Animale and by the Ministère de l′Enseignement Supérieur et de la Recherche (France).

For karyotyping, iPSC clones were grown on Matrigel (BD Biosciences) and treated during their exponential phase of cellular growth with 0.1 µg/ml colcemid (Karyomax Invitrogen Gibco). Karyotyping was done by CHROMOSTEM (CHRU, Montpellier).

### IPS cells differentiation into neural precursor cells

NPCs were generated and subsequently differentiated into neural cells from the iPSC lines as described previously^22^. Briefly, iPSC were mechanically dissociated from feeder-layer and small groups of iPSC were grown in suspension for 48 h in N2B27 pro-neural medium (1:2 DMEM:F12, 1:2 Neurobasal, N2, B27 without vitamin A, 2-mercaptoethanol and gentamicin, Gibco). To further differentiate cells into neuroepithelial (NEP)-rosettes, cells were seeded into 16 μg/ml poly-L-ornithine at (Sigma) and 2 μg/mm laminin (Sigma) (POLAM)-coated glass plates and medium was supplemented with 200 ng/ml Noggin (R&D Systems) and 10 μM SB431542 (Tocris) to induce neuroectoderm cell differentiation as previously described^22,62^. NEP-rosettes appeared between days 8–12, then were mechanically selected and expanded twice each 8–12 days obtaining a neural rosette-enriched culture. Noggin and SB431542 were removed from the medium once NEP-rosettes were formed and during the last round of expansion.

When they reached to confluence NEP-rosettes were differentiated into NPCs. Briefly NEP-rosettes were enzymatically dissociated with 0.05% trypsin/EDTA (Gibco), expanded into POLAM-coated plates in pro-neural medium supplemented with FGF2 at 10 ng/ml and EGF at 10 ng/ml (PeproTech) prompting NPC self-renewal. NPCs were expanded for 5 passages obtaining a homogeneous and phenotypically stable cell population. NPCs were cryopreserved in cryomedium based on 95% CryoStorCS5 (Biolife Solutions) and 5% DMSO (Sigma). NPC self-renewal and ability to generate neurons were retained after thawing.

### iPSC-NPC differentiation into neurons in 2D cultures

For neuronal differentiation, NPCs were plated into POLAM-coated glass plates and cultured in neural induction medium (pro-neural medium supplemented with 10 ng/ml BDNF, 10 ng/ml GDNF, 10 ng/ml NT-3 (all from PeproTech) and 100 µM cAMP (Sigma-Aldrich)). Half of the medium was weekly replaced with fresh media. For immunofluorescence studies cells were plated into POLAM-HCl treated glass coverslips as previously described^63^).

### iPSC-NPC differentiation into neurons in 3D cultures

NPCs were expanded as monolayers and 3D neural differentiation was performed in software-controlled stirred-tank bioreactors (DASGIP, Eppendorf), accordingly to Simão *et al*.^20^. Briefly, NPCs were inoculated as single cells at 4 × 10^5^ cell/ml in pro-neural medium supplemented with 5 ng/ml FGF2, 5 ng/ml EGF and 5 µM Y-27632 ROCK-inhibitor (Merck-Millipore). Cell culture parameters were: pH 7.4, 15% DO and temperature 37 °C. An initial stirring of 70 rpm was used. Perfusion was initiated at day 2 after inoculation, when the cells have aggregated into compact aggregates. A dilution rate of 0.33 day^−1^ was used. The retention of cells in the perfusion was performed using a 20 µm pore stainless steel sparger. Neural differentiation was initiated 1 week after inoculation by changing the medium to neural induction medium and induced for 3 weeks.

### Lysosomal enzymes activities in cell extracts

Enzymatic activities were measured as previously described^64^. Briefly, cells were lysed using 1% Triton X-100, 10% glycerol, 20 mM Tris-HCl pH 7.5 and 150 mM NaCl supplemented with a protease inhibitor cocktail (Roche). Cellular lysates were subjected to three cycles of freeze and thaw and centrifuged for 15 min at 15,000 *g* at 4 °C. Protein content of cell lysate was quantified using the Micro BCA^TM^ Protein Assay Kit (Thermo Scientific). Enzymatic reactions were performed in 48-well plates with 20 μl of protein extracts and 100 μl of reaction buffer (100 mM sodium acetate, pH 4.8 containing 10 mM of enzyme substrate) for 1 h at 37 °C. Substrates for β-gluc and for β-hexosaminidase (β-hex) were 4-methylumbelliferyl-β-d-glucuronide and 4-methylumbelliferyl-N-acetyl-β-d-glucosaminide (both from Sigma-Aldrich), respectively. Enzymatic reactions were stopped by adding 500 μl of 200 mM sodium carbonate pH 10, and fluorescence intensity was measured at 450 nm using Tecan Infinite® M200 Microplate Reader. Standard curves were performed using serial dilutions of 4-methylumbelliferyl (4-MU; Sigma-Aldrich). Enzyme activity is expressed in nmol 4-MU/μg of protein/h.

### Immunofluorescences

For indirect immunofluorescence, NPCs and neurons were fixed with 4% PFA (EM grade, PKS) in PBS for 15 min at RT and permeabilized with 0.1% Triton X-100 in PBS at RT for 5 min. Cells were then blocked in blocking solution (BS): 2% bovine serum albumin (Sigma) and 10% horse serum in PBS for 1 h at RT. Primary antibodies were diluted in BS and incubated overnight at 4 °C, followed by incubation with secondary antibodies (Alexa Fluor series from Invitrogen) all diluted 1:500 in BS for 1 h at RT. Samples were then mounted with fluorescent mounting medium (Dako) containing DAPI at 10 ng/μl (Sigma). Primary antibodies used are: SSEA-3, TRA-2-49, NANOG, α-fetoprotein, smooth muscle actin, TUJ1, Nestin, Sox2, OTX1-2, GFAP, LAMP1, CAR, NeuN, Tbr1, Brn2, vGLUT, and PSD95. Phalloidin Red (Sigma) was used at 1:500 in BS, 1 hour at RT.

Images were taken using Leica LSM780 and Zeiss SP5 confocal microscopes, with 20X/0.8 Plan-Apo, 40X/1.3 oil-DIC Plan-Apo or 63X/1.4 oil-DIC Plan-Apo objectives. Image analysis was performed using Fiji Software^65^.

### Acidic vesicles and LAMP1^+^ vesicles quantification

NPCs plated into POLAM-coated glass bottom dishes (Wilco) were incubated with Lysotracker^TM^ (Invitrogen) at 1 nM for 1 hour at 37 °C. Probe-containing medium was replaced by fresh medium before imaging. Images were acquired using a Zeiss LSM780 confocal microscope with 63X/1.4 oil-DIC Plan-Apo objective with ZEN software. For acidic vesicle quantification 10 images containing 7–10 cells each were taken. Particle size was analyzed in 80–90 cells using Fiji software. Results are expressed as lysosomes/cell (0.1–1.2 μm^2^).

### LAMP1^+^ vesicles quantification

For the quantification of LAMP1-positive vesicles and LAMP1 immunoreactive area, 10 neurons (TUJ1^+^) per clone and time point were imaged using a Leica SP5 confocal microscope. Acquisition was performed with a resolution of 12.01, 12.01, 125.9 nm/pixel (in x, y, z, respectively). Blurring (due to diffraction limited imaging by the confocal) and noise (due to photon noise) were reduced by image deconvolution using Huygens Essential 3.7 (Scientific Volume Imaging). Quantification of LAMP1^+^ vesicles and LAMP1-immunoreactive areas were made using Fiji software^65^. First, neuronal soma on each image was manually selected. Then lysosomes were obtained by image processing using FeatureJ-Laplacian plug in (http://www.imagescience.org/meijering/software/featurej/) and segmentation by Triangle thresholding^66^.

### Glycosaminoglycans quantification

Aggregates were collected at days 7 and 28 of culture and lysed in papain extraction reagent (0.2 M sodium phosphate buffer, pH 6.4, containing 100 mM sodium acetate, 5 mM cysteine, 5 mM EDTA, 0.1 mg/ml papain (Sigma-Aldrich)) for 3 h at 65 °C. After centrifugation at 10,000 g for 5 min., sulfated GAG content was determined in the supernatants using the Blyscan Sulfated Glycosaminoglycan Assay (Biocolor) according to manufacturer’s instructions.

### Protein extraction and western blotting

For protein extraction, cells were washed once with PBS and incubated with lysis buffer (1% Triton X-100, 10% glycerol, 20 mM Tris-HCl pH 7.5 and 150 mM NaCl) supplemented with protease inhibitor cocktail (Roche) for 10 min at 4 °C. Cellular lysates were centrifuged for 15 min at 14,000 rpm at 4 °C. Supernatants of total protein extracts were quantified using BCA (Pierce) or Bradford reagent (BioRad). Proteins were denatured in SDS-containing loading buffer for 10 min at 95 °C. Proteins were separated in SDS-PAGE and transferred to PVDF membrane. The membrane was probed with anti**-**p62, anti-LC3-I/II, anti-β-tubulin, anti-EGFR, anti-CAR and anti-actin antibodies.

### RNA extraction and qRT-PCR analysis

Real-time quantitative PCR analysis (RT-qPCR) was performed as described in Brito *et al*.^67^. Briefly, total RNA was extracted with High Pure RNA Isolation kit (Roche) and quantified using a NanoDrop 2000c (Thermo Scientific). Reverse transcription was performed with Transcriptor High Fidelity cDNA Synthesis kit (Roche), using anchored-oligo(dT)18 primer. qRT-PCR analysis was performed in a LightCycler 480 (Roche) according to Light-Cycler 480 SYBR Green I Master Kit (Roche) instructions. Primers were used at 5 µM, in 20 µL reactions; each sample was performed in triplicates. See Table 1 for list of primers. Cycles threshold (Ct’s) and melting curves were determined using LightCycler 480 software, version 1.5 (Roche), and results were processed using the 2^−ΔΔCt^ method for relative gene expression analysis^67,68^. Changes in gene expression were normalized using the housekeeping gene RPL22 (coding for ribosomal protein L22) as internal control.

**Table 1:** Primer sequences.

Target	Forward (5′ → 3′)	Reverse (5′ → 3′)
*SOX2*	GGGAAATGGGAGGGGTGCAAAAGAGG	TTGCGTGAGTGTGGATGGGATTGGTG
*OCT4*	GACAGGGGGAGGGGAGGAGCTAGG	CTTCCCTCCAACCAGTTGCCCCAAAC
*NANOG*	CAGCCCCGATTCTTCCACCAGTCCC	CGGAAGATTCCCAGTCGGGTTCACC
*LIN28*	AGAGTAAGCTGCACATGGAAGGGT	TATGGCTGATGCTCTGGCAGAAGT
*trSOX2*	TAAAGCAGCGTATCCACATAGC	GCCATTAACGGCACACTGC
*trOCT4*	TAAAGCAGCGTATCCACATAGC	TCACCACTCTGGGCTCTC
*cMYC*	TAAAGCAGCGTATCCACATAGC	CGGAAACGACGAGAACAGTTG
*trKLF4*	TAAAGCAGCGTATCCACATAGC	ACCACCTCGCCTTACACATG
*hMYC*	GCGTCCTGGGAAGGGAGATCCGGAGC	TTGAGGGGCATCGTCGCGGGAGGCTG
*PCNA*	CGGAGTGAAATTTTCTGCAAG	TTCAGGTACCTCAGTGCAAAAG
*nestin*	TAAGGTGAAAAGGGGTGTGG	GCAAGAGATTCCCTTTGCAG
*βIII-tubulin*	GGGCCTTTGGACATCTCTTC	CCTCCGTGTAGTGACCCTTG
*Synaptophysin (SYP)*	TTTGTGAAGGTGCTGCAATG	GCTGAGGTCACTCTCGGTCT
*VGluT-1*	GTTCTGCTGCTCGTCTCCT	ATGAGTTTCGCGCTCTCTCC
*TH*	AGCCCTACCAAGACCAGACG	GCGTGTACGGGTCGAACTT
*GAD67*	ACCAGAAAACTGGGGCTCAA	GCAGGTTCTTGGAGGATTGC
*GFAP*	AGAGAGGTCAAGCCAGGAG	GGTCACCCACAACCCCTACT
*GLT-1*	CCAGGAAAAACCCCTTCTCC	TCTTCCAGGCAACGAAAGGT
*GUSB*	GGCTACCTCCCCTTCGAG	GTTGAAGAACTGCGGCAGC
*RPL22*	CACGAAGGAGGAGTGACTGG	TGTGGCACACCACTGACATT
*GAPDH*	AGAACATCATCCCTGCCTCT	ACCCTGTTGCTGTAGCCAAA

### Cell treatment for protein degradation assays

NPCs at 80% of confluence were incubated with 200 ng/ml EGF (PeproTech) or 200 ng/ml FK^CAV^ for 15 min at 4 °C allowing ligand-receptor binding. To synchronize ligand-receptor internalization, ligand-containing medium was replaced by fresh pre-warmed medium. Then cells were incubated at 37 °C allowing ligand-receptor internalization. At indicated times proteins were extracted and subjected to western blotting.

### Ca^++^ fluorescence imaging

For calcium fluorescence imaging experiments in 2D cultures, neuronal cultures were grown on glass coverslips coated with POLAM to prevent cells detaching or moving during imaging experiments. Prior to imaging, cells were loaded with 5 µM acetoxy-methyl-ester Fura-2 (Fura-2 AM, Invitrogen) for 45 min in Tyrode solution (physiological saline) containing 0.5% Pluronic acid at 37 °C. Imaging experiments were performed at 22 °C using an IX70 Nikon microscope equipped with a X20 objective. The cultures were excited alternatively at 340 and 380 nm and light emission at 510 nm was used to determine the fluorescence ratio (F340/F380) that reflect the intracellular calcium concentration. Images were acquired every 3 seconds for 10 to 15 min. Calcium signals attributable to neuronal cells were measured by switching the standard Tyrode solution to a 60 mM KCl/Tyrode solution to depolarize the cells and to activate voltage-dependent calcium channels. 1,4-dihydropyridine-derivative (5 µM) (Nimopidine (Alomone Labs) was used to specifically block L-type calcium channels, a marker of neuronal activity. All the imaged cultures were ultimately treated with 10 µM ionomycin (Sigma) to validate cell viability. Image analysis was performed using MetaFluor Imaging software (Molecular Devices) using the region of interest (ROI) analysis method. Three neuronal cultures/neuron sources were analyzed.

For calcium imaging experiments in 3D cultures, aggregates were collected after 3 weeks of differentiation and incubated with 1x Fluo-4 Direct^TM^ calcium reagent (Invitrogen) for 30 min at 37 °C, 5% CO_2_, and 3% O_2_, followed by 15 min at RT. Samples were then imaged live using a spinning-disk confocal microscope (Nikon Eclipse Ti-E, confocal scanner: Yokogawa CSU-x1). Image analysis was performed using FluoroSNNAP – Fluorescence Single Neuron and Network Analysis Package, an open-source software developed in MatLab for automated quantification of calcium dynamics of single cells and network activity patterns^31^.

### Synaptic vesicle trafficking assay

Synaptic vesicles trafficking assays were based on^69^. Aggregates were plated in POLAM-coated glass coverslips and incubated with 5 mM HEPES-NaOH, pH 7.4; 10 mM glucose; 2.5 mM CaCl_2_; 1 mM MgCl_2_; 100 mM KCl; 37 mM NaCl (referred to as 100 mM KCl buffer) for 5 min. Afterwards, 100 mM KCl buffer was removed and aggregates incubated with 10 M FM-1–43 dye (Invitrogen) in 5 mM HEPES-NaOH, pH 7.4; 10 mM glucose; 2.5 mM CaCl_2_; 1 mM MgCl_2_; 5 mM KCl; 37 mM NaCl (referred to as 5 mM KCl buffer) for 15 min. Aggregates were washed for 1 min with 5 mM KCl buffer with ADVASEP-7 (Sigma), followed by three washes with 5 mM KCl buffer. Exocytosis was stimulated with 100 mM KCl buffer and samples were visualized live using a fluorescence microscope (Leica DMI6000) in order to monitor the decreasing of fluorescence intensity overtime. Fluorescence intensity was measured using ImageJ software version 1.47q (http://rsbweb.nih.gov/ij/).

### TEM on ultrathin sections and toluidine blue stained thin sections

Neurons were differentiated from NPCs and grown for 6 and 9 weeks in 60 mm POLAM-coated glass dishes as described above. Cells were fixed with 2.5% glutaraldehyde in cacodylate buffer (0.1 M pH 7.1–7.4) for 2 h at 4 °C and washed three times with cacodylate buffer (0.1 M pH 7.1–7.4). Then cells were collected and treated with 1% osmium tetroxide in cacodylate buffer (0.1 M pH 7.1–7.4) for 2 h at room temperature, dehydrated with ethanol and embedded in EPON. Thin (250–1000 nm) and ultrathin (80 nm) sections were obtained with ultra-microtome Leica EM UC6. 500–1000 nm-thick sections were stained with toluidine blue and images were acquired with Leica DM6000B upright microscope. Ultrathin sections were then contrasted with uranyl acetate. Images were acquired with JEOL-JEM-1011. Images were analyzed using ImageJ software.

### Experimental Design and Statistical analysis

Data are mean ± SD of, at least, 3 independent experiments and were analyzed with GraphPad Prism 6 software. One-way ANOVA followed by Tukey’s post-hoc multiple comparison analysis was used (*p < *0.05*, ***p < *0.01*, ***p < *0.001*).

For 3D cultures, analysis of neurospheroids from different stirred-tank bioreactor cultures are considered biological replicates, while analysis of different neurospheroids sampled from the same bioreactor culture are considered technical replicates.

## Electronic supplementary material

Supplemental figure 1
