# Supplementary material for: Lysosomal and network alterations in human mucopolysaccharidosis type VII iPSC-derived neurons
Source: Sci Rep. 2018 Nov 9;8:16644. doi: 10.1038/s41598-018-34523-3 (PMC6226539; doi:10.1038/s41598-018-34523-3)
Supplement: Supplementary file 1 — Supplemental figure 1 [file 41598_2018_34523_MOESM1_ESM.pdf]

Lysosomal and network alterations in human mucopolysaccharidosis type VII iPSC-derived neurons

Neus Bayó-Puxan[1,2,*], Ana Paula Terrasso[3,4,*], Sophie Creyssels[1,*], Daniel Simão[3,4], Christina Begon-Pescia[1], Marina Lavigne[1], Sara Salinas[1], Florence Bernex[5], Assumpció Bosch[6], Vasiliki Kalatzis[7], Thierry Levade[8], Ana Maria Cuervo[9], Philippe Lory[10], Antonella Consiglio [2,11,#], Catarina Brito[3,4,#,°] & Eric J Kremer[1,#]

Supplemental Information

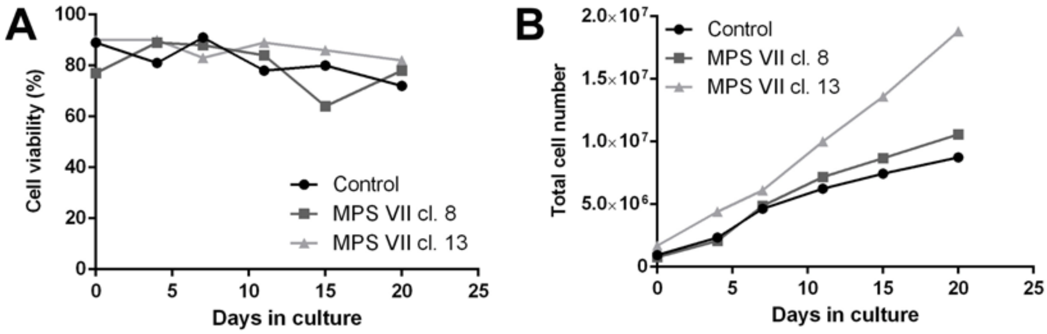

Supplemental Figure 1. Viability and self-renewal capacity of iPSC-NSCs

(a) Cell viability along culture time; days in culture corresponding to cell passages 12 to 18. **(b)** Cumulative cell number along culture time; days in culture corresponding to cell passages 12 to 18.
